# Effect of free oxygen radical anions and free electrons in a Ca_12_Al_14_O_33_ cement structure on its optical, electronic and antibacterial properties

**DOI:** 10.1016/j.heliyon.2019.e01808

**Published:** 2019-05-30

**Authors:** Chaiwat Phrompet, Chaval Sriwong, Pornjuk Srepusharawoot, Santi Maensiri, Prinya Chindaprasirt, Chesta Ruttanapun

**Affiliations:** a Smart Materials Research and Innovation Unit (SMRIU), Faculty of Science, King Mongkut's Institute of Technology Ladkrabang, Chalongkrung Road, Ladkrabang, Bangkok 10520, Thailand; b Department of Physics, Faculty of Science, King Mongkut's Institute of Technology Ladkrabang, Chalongkrung Road, Ladkrabang, Bangkok 10520, Thailand; c Department of Chemistry, Faculty of Science, King Mongkut's Institute of Technology Ladkrabang, Chalongkrung Road, Ladkrabang, Bangkok 10520, Thailand; d Institute of Nanomaterials Research and Innovation for Energy (IN-RIE), Department of Physics, Faculty of Science, Khon Kaen University, Khon Kaen 40002, Thailand; e School of Physics, Institute of Science, Suranaree University of Technology, Nakhon Ratchasima 30000, Thailand; f Thailand Center of Excellence in Physics, Commission on Higher Education, 328 Si Ayutthaya Road, Bangkok 10400, Thailand; g Sustainable Infrastructure Research and Development Center, Department of Civil Engineering, Faculty of Engineering, Khon Kaen University, Khon Kaen 40002, Thailand; h Academy of Science, The Royal Society of Thailand, Dusit, Bangkok 10300, Thailand

**Keywords:** Materials science, Civil engineering, Condensed matter physics, Materials chemistry, Bioengineering, Free oxygen radical anions, Optical property, Electronic property, Antibacterial property, Ca_12_Al_14_O_33_ cement

## Abstract

The aim of this work was to investigate the effect of free oxygen radicals and free electrons in a Ca_12_Al_14_O_33_ (C12A7) cement structure on the optical, electronic and antibacterial activity of this material. Ca_12_Al_14_O_33_ was successfully fabricated via rapid heating to high temperatures by high frequency electromagnetic induction. Ca_12_Al_14_O_33_ cement samples were characterized using XRD and UV-Vis-DRS spectroscopy. The morphology and chemical composition of the samples were also investigated using SEM and EDS techniques. The presence of free oxygen radicals (O_2_^−^ions) in the insulating structure of Ca_12_Al_14_O_33_ was confirmed using Raman spectroscopy showing a spectrum peak at 1067 cm^−1^. The excitation of free electrons in the Ca_12_Al_14_O_33_ cement was indicated by UV-Vis absorption spectra at 2.8 eV and an optical energy gap of 3.5 eV, which is consistent with the first-principles calculations for the band energy level. The effects of free oxygen radicals and free electrons in the Ca_12_Al_14_O_33_ structure as antibacterial agents against *Escherichia Coli (E*. *coli)* and *Staphylococcus Aureus (S*. *aureus)* were investigated using an agar disk-diffusion method. The presence of O_2_^−^ anions as a reactive oxygen species (ROS) at the surface of Ca_12_Al_14_O_33_ caused inhibition of *E*. *coli* and *S*. *aureus* cells. The free electrons in the conducting C12A7 reacted with O_2_ gas to produce ROS, specifically super oxides (O_2_^−^), superoxide radicals (O_2_^•-^), hydroxyl radicals (OH^•^) and hydrogen peroxide (H_2_O_2_), which exhibited antibacterial properties. Both mechanisms were active against bacteria without effects from nano-particle sized materials and photocatalytic activity. The experimental results showed that the production of ROS from free electrons was greater than that of the free O_2_^−^ anions in the structure of Ca_12_Al_14_O_33_. The antibacterial actions for insulating and conducting Ca_12_Al_14_O_33_ were different for *E*. *coli* and *S*. *aureus*. Thus, Ca_12_Al_14_O_33_ cement has antibacterial properties that do not require the presence of nano-particle sizes materials or photocatalysis.

## 1 Introduction

Currently, the use of antimicrobials to promote public health is a research topic with much attention [1, 2, 3, 4]. The goal of such research is to identify materials with antibacterial properties capable of inhibiting or killing various bacteria through mechanisms not limited to photocatalytic and nano-particle effects [4,5]. Antibacterial materials have attracted significant attention due to their very interesting application in preventing bacterial growth on smart building bathroom and kitchen walls [4]. There are four types of antibacterial activity: (1) cation elution, (2) pH effects, (3) electrostatic interactions between the surfaces of bacteria with nano-size particles, and (4) the effects of active oxygen species [4,6,7]. In addition to these, photocatalytic activity, electrostatic interaction, cellular internalization of nanoparticles and production of reactive oxygen species (ROS) also have antimicrobial activity [4, 7, 8, 9]. ROS effects are predominantly used for antibacterial activity since it is an easy mechanism to employ [7,8]. ROS are comprised of hydroxyl radicals (OH^•^), hydrogen peroxide (H_2_O_2_) and superoxide ions (O_2_^-^) [4,8]. These materials cause death by contacting bacterial membranes and directly damaging their surfaces [4,7]. ROS species can be generated from the reaction of free particles (free electrons and holes) in the course of photocatalytic activities [9, 10, 11]. The photocatalytic effect causes generation of free electrons and holes in materials using phonon energy corresponding to energy gap of the materials. There have been many reports that titanium dioxide (TiO_2_) [12] and zinc oxide (ZnO) [10] can generate ROS through photocatalysis. Practically, TiO_2_ and ZnO used phonon energy at 3.1 eV [13] and 3.2 eV [7], respectively, to photocatalytically produce ROS. They are produced from O_2_ and H_2_O adsorption promoting a reaction of free electrons and holes at the surfaces of materials to produce OH^−^, H_2_O_2_ and O_2_^-^. Alternatively, ZnO [7, 8, 9,14] nanoparticles cause death of bacterial cells through electrostatic interactions between bacterial surfaces with nanoparticles in the absence of light, thereby damaging cell membranes. TiO_2_ and ZnO act through photocatalysis and nanoparticle effects to produced antibacterial activity.

**Fig. 1:**
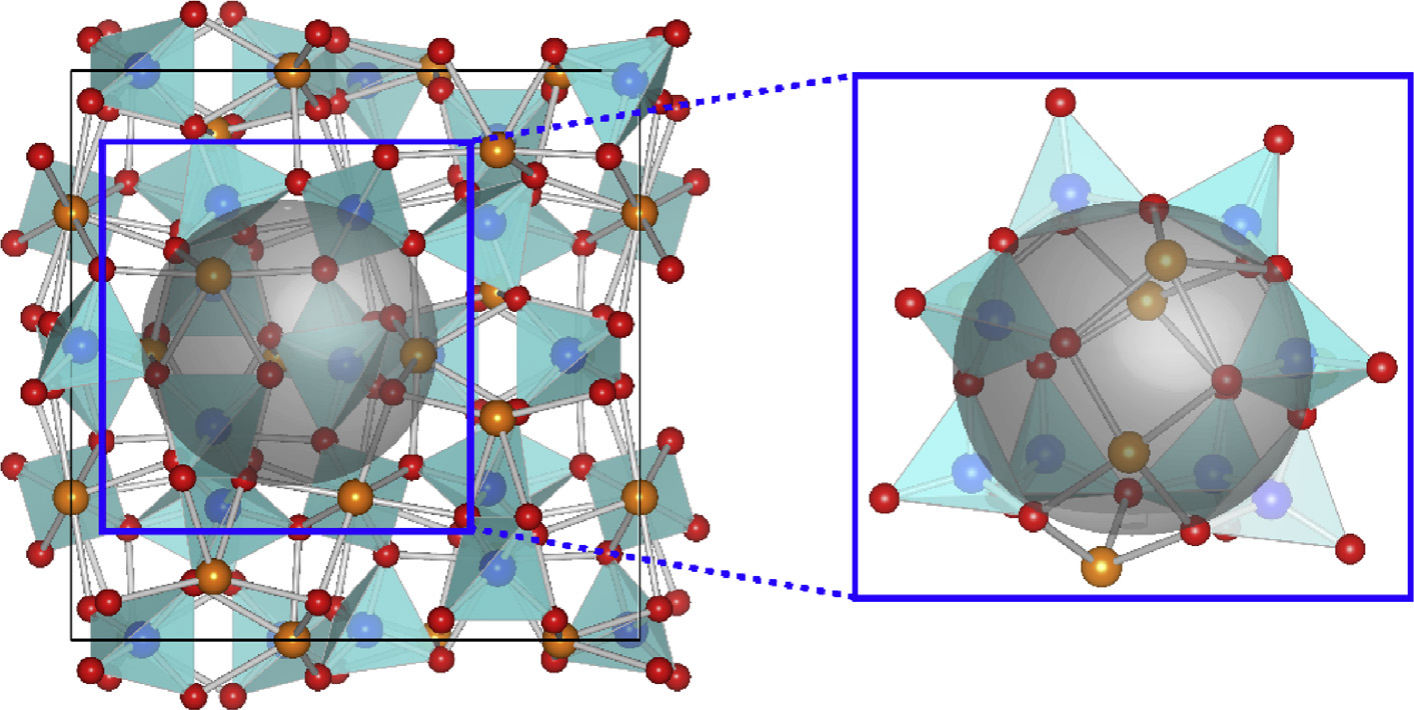
A unit cell of the Ca_12_Al_14_O_33_ cement structure: blue, orange and red balls denote Al, Ca and O atoms, respectively. The grey sphere represents the cage of this structure. The crystal structure of Ca_12_Al_14_O_33_ contains nano-cages with a set inner diameter.

Recently, it has been reported that Ca_12_Al_14_O_33_ cement exhibits free oxygen radical O^−2^ ions in a vacant cage structure [15, 16, 17,18]. The Ca_12_Al_14_O_33_ cement structure is linked by calcium, aluminum and oxygen atoms forming empty nanometer-sized cages within the structure [19,20], as shown in Fig. 1. A unit cell of insulating Ca_12_Al_14_O_33_ is comprised of two molecules occupying 12 crystallographic nano-cages while presenting a 4^+^ charge at the cage wall, represented as [Ca_24_Al_28_O_66_]^4+^ [21]. The two cages in this unit cell support electrical neutrality by entrapping two free oxygen ions (O^−2^) in cages referred to as an extra-framework [21]. The Ca_12_Al_14_O_33_ structure was revealed to contain free O^−2^ ions (as replaced by free oxygen radicals, O^−^, O_2_^-^, or O_2_^2-^) loosely bound to a lattice framework, represented as Ca_12_Al_14_O_33_:O^−2^ [21,22]. Hayashi et al. [23] showed that the process of preparing Ca_12_Al_14_O_33_ cement in a dry oxygen atmosphere at temperature 1350 °C could produce both O^−^ and O_2_^-^ (as ROS species). Lu et al. [24] reported that the present of O^−^ and O_2_^-^ resulted in antibacterial activity. Nevertheless, antimicrobial materials should be effective under various conditions and not limited to photocatalytic activity or nano-particle effects. Furthermore, Hayashi et al. [23] reported that free O^−^ and O_2_^-^ could be replaced by free electrons in a conducting Ca_12_Al_14_O_33_ cement, represented as Ca_12_Al_14_O_33_:e^−^. It has been reported that this material has distinct optical and electrical properties. However, to best of our knowledge, there are no reports of the effect and mechanisms of free electrons in the nano-cage structure of Ca_12_Al_14_O_33_ cement on antibacterial activity.

This work aims to investigate the effect of free electrons in the nano-cage structure of Ca_12_Al_14_O_33_ cement on the optical, electronic and antibacterial activities of these materials. The developed material presenting free electrons in a nano-cage structure was characterized. Conducting Ca_12_Al_14_O_33_ cement with free electrons was rapidly prepared by heating insulator cement inside a carbon crucible at high temperatures using high frequency electromagnetic induction. The mechanism and effect of free electrons and free oxygen radicals in the nano-cage structure of these materials on its optical, electronic and antibacterial properties were also investigated. Their antibacterial activities against gram-negative *E*. *coli* and gram-positive *S*. *aureus* are reported. Moreover, the mechanism of the antibacterial action of free electrons and free oxygen radicals in the nano-cage structure of Ca_12_Al_14_O_33_ cement is described.

## 2 Experimental

### 2.1 Chemicals

Calcium carbonate (CaCO_3_, 99% Sigma-Aldrich), alumina powder (Al_2_O_3_, 99.9% Sigma-Aldrich) and ethanol (95%) were used as the starting raw materials. All chemicals were used as received with no further purification.

#### 2.1.1 Preparation of Ca_12_Al_14_O_33_ cement as starting materials

Ca_12_Al_14_O_33_ cement powder was first prepared via a solid-state reaction using CaCO_3_ and Al_2_O_3_ as starting materials. In brief, CaCO_3_ and Al_2_O_3_ powders were stoichiometrically prepared following the reaction, 12CaCO_3_ + 7Al_2_O_3_ → Ca_12_Al_14_O_33_ + 12CO_2_. The powders were mixed by ball milling in ethanol for 24 h at room temperature, and oven-drying at 100 °C for 24 h. After that, the obtained powder was placed in an alumina crucible, and then sintered in an electric furnace at 1200 °C under air atmosphere for 24 h. Finally, the obtained Ca_12_Al_14_O_33_ sample, referenced as CAO@1200C, was crushed into a powder and used as a starting material to synthesize a conducting Ca_12_Al_14_O_33_ cement sample.

#### 2.1.2 Preparation of conducting Ca_12_Al_14_O_33_ cement

The as-prepared CAO@1200C powder was used as a starting material for fabrication of conducting Ca_12_Al_14_O_33_ cement. A mass of 100 g of CAO@1200C powder was placed in a carbon crucible with a carbon cap. Then, the carbon crucible was transferred into the middle of a Cu induction coil. High frequency electromagnetic induction heating was done using an induction coil (Model: TH-60AB (90 A, 3 phase, 380 V, 50–60 kHz)). The temperature was determined using an IR detector (Model: SENTEST (NS50PH1FF), accuracy class:2.0) focused on the surface of the carbon crucible. The CAO@1200C powder was rapidly heated from room temperature to sintering temperatures of 1350 °C, 1450 °C and 1550 °C with a 40 sec holding time (referenced as the CAO@1350C, CAO@1450C and CAO@1550C samples, respectively). Finally, the samples were cooled by natural convection to room temperature.

#### 2.1.3 Preparation of the cement pellets

For pellet fabrication, the obtained CAO@1350C, CAO@1450C and CAO@1550C powders were subjected to uniaxial compression and pressed into disc-shaped pellets that were 10 mm in diameter and 2–3 mm thick. Then, the antibacterial activity of these pellets was tested.

### 2.2 Characterization

The lattice parameters were determined using an X-ray diffractometer (XRD), (Rigaku, Miniflex Cu K-alpha radiation), with a 2θ scanning range from 10 to 80^o^ and step interval of 0.02^o^. Absorption spectroscopy was also done using a UV-Vis Spectrometer (Perkin Elmer, Lamda 950). A scanning electron microscope (SEM), JSM5800LV, JEOL, Japan with energy dispersive X-ray spectroscopy (EDX) (Oxford ISIS 300) was used to measure and confirm the morphologies of all the cement particles and bacteria, along with the elemental composition of the cement samples.

### 2.3 First-principles calculations

A first-principles approach was employed with the density of states of Ca_12_Al_14_O_33_:2O^2-^ cement and Ca_12_Al_14_O_33_:4e^−^ cement using the Vienna *Ab initio* Simulation Package (VASP) [25]. The pseudopotential used in this work was based on the Projector Augmented Wave (PAW) approach [26]. The PAW valence states were 3s and 3p, 4s, 3s and 3p, and 2s and 2p for Ca, Al and O, respectively. In this work, the Ceperley-Alder form of the exchange-correlation functional [27], which is the local density approximation (LDA), was used to determine the electronic density of states of both the Ca_12_Al_14_O_33_:2O^2-^ and Ca_12_Al_14_O_33_:4e^−^ cements. A 600 eV plane-wave cutoff energy and 5 × 5 × 5 K-point sampling of the Brillouin zone were used for all calculations. The HSE06 hybrid functional was chosen to determine the density of the Ca_12_Al_14_O_33_:4e^−^ states.

### 2.4 Property measurements

The vibration mode of atomic bonding was evaluated using Fourier-transform infrared spectroscopy (FTIR), (Bruker, Senterra). The optical properties of the samples were investigated using a diffused reflectance UV-Visible spectrometer, (DRS) (Perkin Elmer, Lambda 950). Optical measurements were used to determine the absorption coefficient spectra of the specimens at room temperature.

### 2.5 Antibacterial property testing

The antibacterial properties of the CAO@1350C, CAO@1450C and CAO@1550C samples were tested using an agar disk-diffusion method against a gram-negative bacterium, *Escherichia coli (E*. *coli)* (ATCC 25922) and a gram-positive bacterium, *Staphylococcus aureus* (*S*. *aureus*) (ATCC 25923). All samples were pellet shaped with diameter of 10 mm. *E*. *coli* and *S*. *aureus* were cultivated on Muller Hinton agar at 37 °C for 24. Then, cells of *E*. *coli* and *S*. *aureus* were suspended in a 0.85%NaCl solution and the cell suspension was adjusted to 0.5 McFarland (1 × 10^8^ CFU/mL). Subsequently, the *E*. *coli* and *S*. *aureus* cell suspensions were swabbed onto Muller Hinton agar. After drying, CAO@1350C, CAO@1450C and CAO@1550C sample pellets were placed on the agar surfaces. Then, the agar plates were incubated (Contherm, Scientific Ltd., New Zealand) at 37 °C for 24 h in a dark incubation chamber. Finally, the inhibition zones on the agar plates were photographed and the widths of inhibition zones reported. Inhibition of *E*. *coli* and *S*. *aureus* was confirmed using scanning electron microscopy (SEM), JSM5800LV, JEOL, Japan.

## 3 Results and discussion

### 3.1 Characterization of Ca_12_Al_14_O_33_ cement as a starting material

**Fig. 2:**
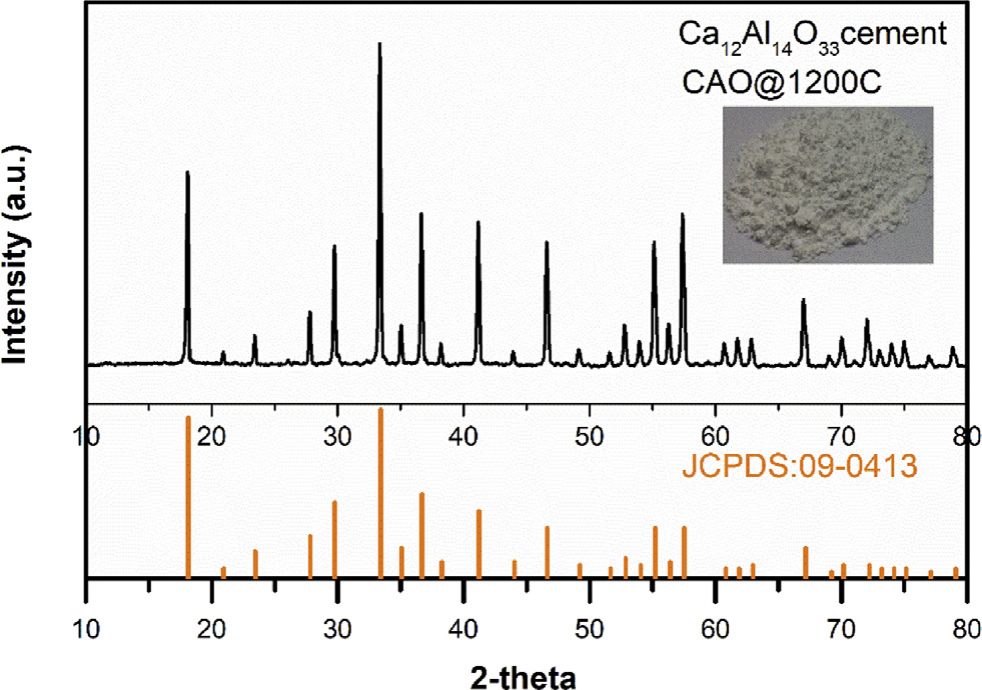
XRD patterns of the CAO@1200C cement sample referencing the JCPDS#09–0413 file of the standard Ca_12_Al_14_O_33_ cement phase.

The CAO@1200C sample was prepared via a solid-state reaction at 1200 °C for use as a starting material for fabrication of conducting Ca_12_Al_14_O_33_ cement. Fig. 2 displays the XRD patterns of the CAO@1200C sample. These XRD results show a pattern corresponding to the JCPDS#09–0413 file (a standard Ca_12_Al_14_O_33_ cement phase). The results confirmed that the prepared CAO@1200C sample formed a phase of Ca_12_Al_14_O_33_ cement.

### 3.2 Characterization of conducting Ca_12_Al_14_O_33_

#### 3.2.1 Sintering process

**Fig. 3:**
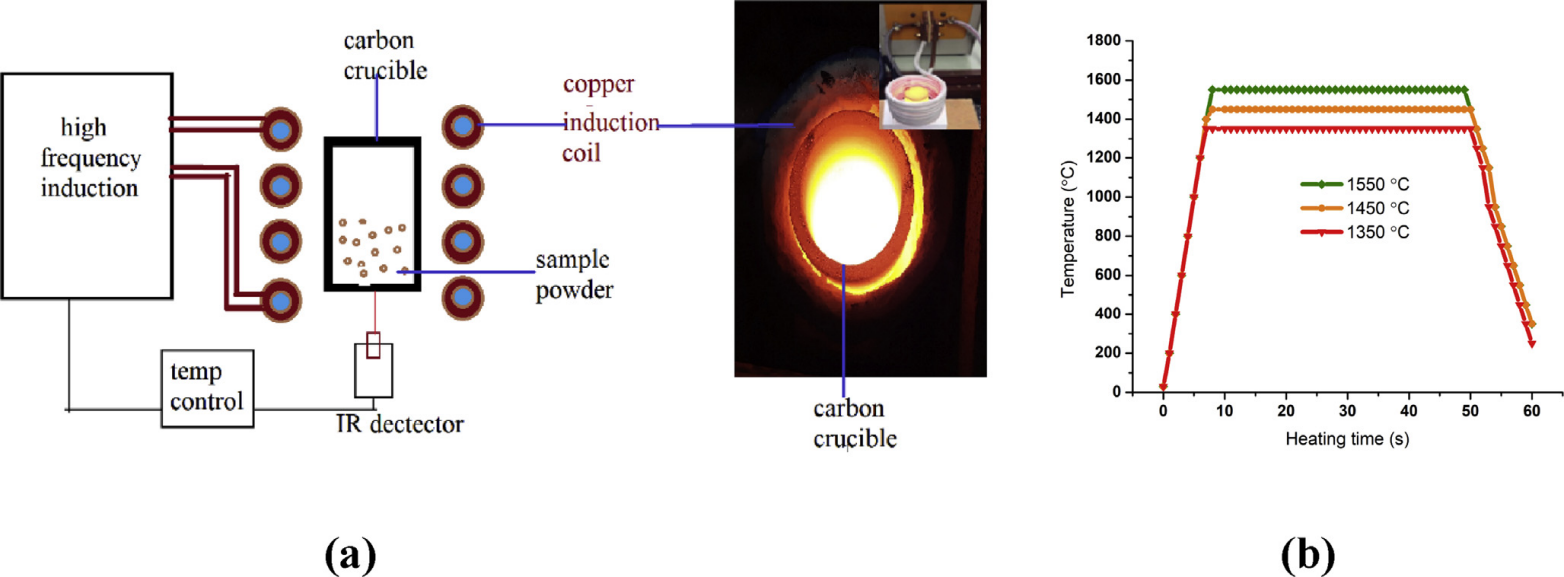
**(a)** Schematic structure of the high frequency induction heating system with an image of material undergoing induction heating and **(b)** the temperature and heating time in the sintering process.

Fig. 3 (a) presents a schematic of an electromagnetic induction heating system for synthesizing the samples fabricated in the course of this work. The starting CAO@1200C powder was loaded into a carbon crucible. The carbon crucible was wrapped with a Cu induction coil. Cooling water was circulated in a Cu coiled tube to protect against overheating on opration of themagnetic induction heating. An IR detector was used to measure the temperature of the carbon crucible and this information used as feedback to control the power of the induction heater. The carbon crucible was rapidly heated from room temperature to 1350 °C, 1450 °C and 1550 °C, with a holding time of 40 seconds for sintering. Then, the samples were rapidly cooled to room temperature. Fig. 3 (b) shows an electromagnetic induction heating time of approximately 1 minute for sintering.

#### 3.2.2 XRD characterization

**Fig. 4:**
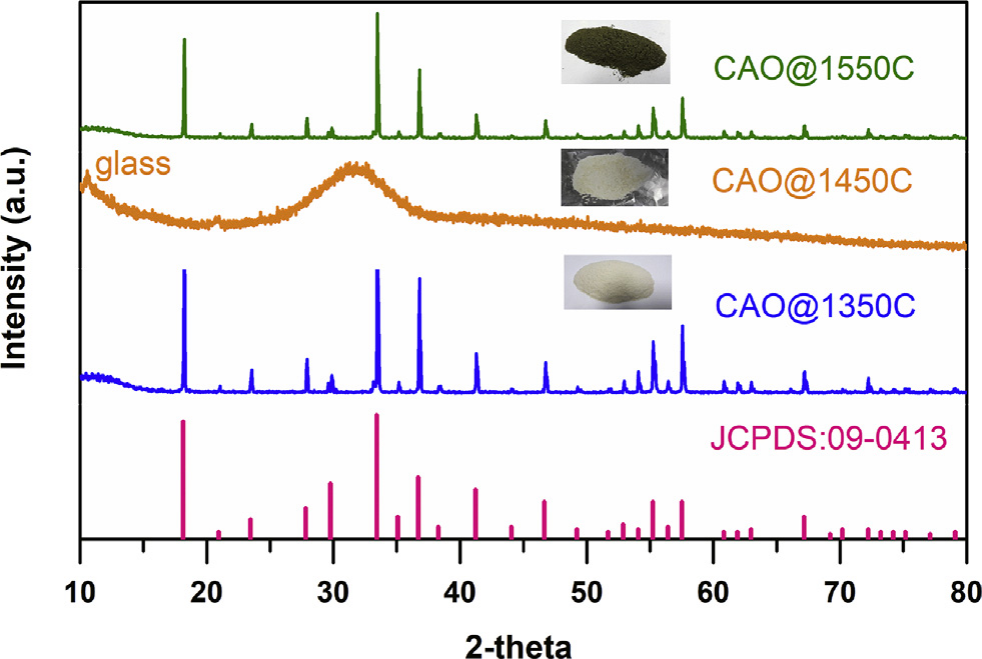
XRD patterns of the synthesized CAO@1350C, CAO@1450C and CAO@1550C samples referencing the JCPDS#09–0413 file.

Fig. 4 shows the XRD patterns of the synthesized CAO@1350C, CAO@1450C and CAO@1550C samples prepared by heating in a carbon crucible by high frequency electromagnetic induction. The XRD results showed that the patterns of the sintered CAO@1350C and CAO@1550C samples matched the JCPDS#09–0413 file as Ca_12_Al_14_O_33_ cement phase [28, 29, 30, 31, 32]. These results confirmed the synthesis of a Ca_12_Al_14_O_33_ cement phase in the CAO@1350C and CAO@1550C samples. In contrast, the XRD patterns of the synthesized CAO@1450C sample exhibited a different XRD pattern pattern that matched the JCPDS#09–0413 file. This result was due to formation of a glass Ca_12_Al_14_O_33_ cement phase as corresponding to that observed in a previously published XRD pattern [33]. This implied that the preparation process produced a glass phase of Ca_12_Al_14_O_33_ cement at a temperature of 1350 °C. Thus, this confirmed that all samples formed phases of Ca_12_Al_14_O_33_ cement during high frequency induction heating. However, the CAO@1350C, CAO@1450C and CAO@1550C samples did not exhibit 2θ XRD peaks at 26.5°, 10.8° and 25.2°. These reference graphite, graphene oxide, and reduced graphene oxide, respectively. These results confirmed that graphite from the crucible did not dissolve into the samples [34].

#### 3.2.3 Photographic and electric resistance measurements

**Fig. 5:**
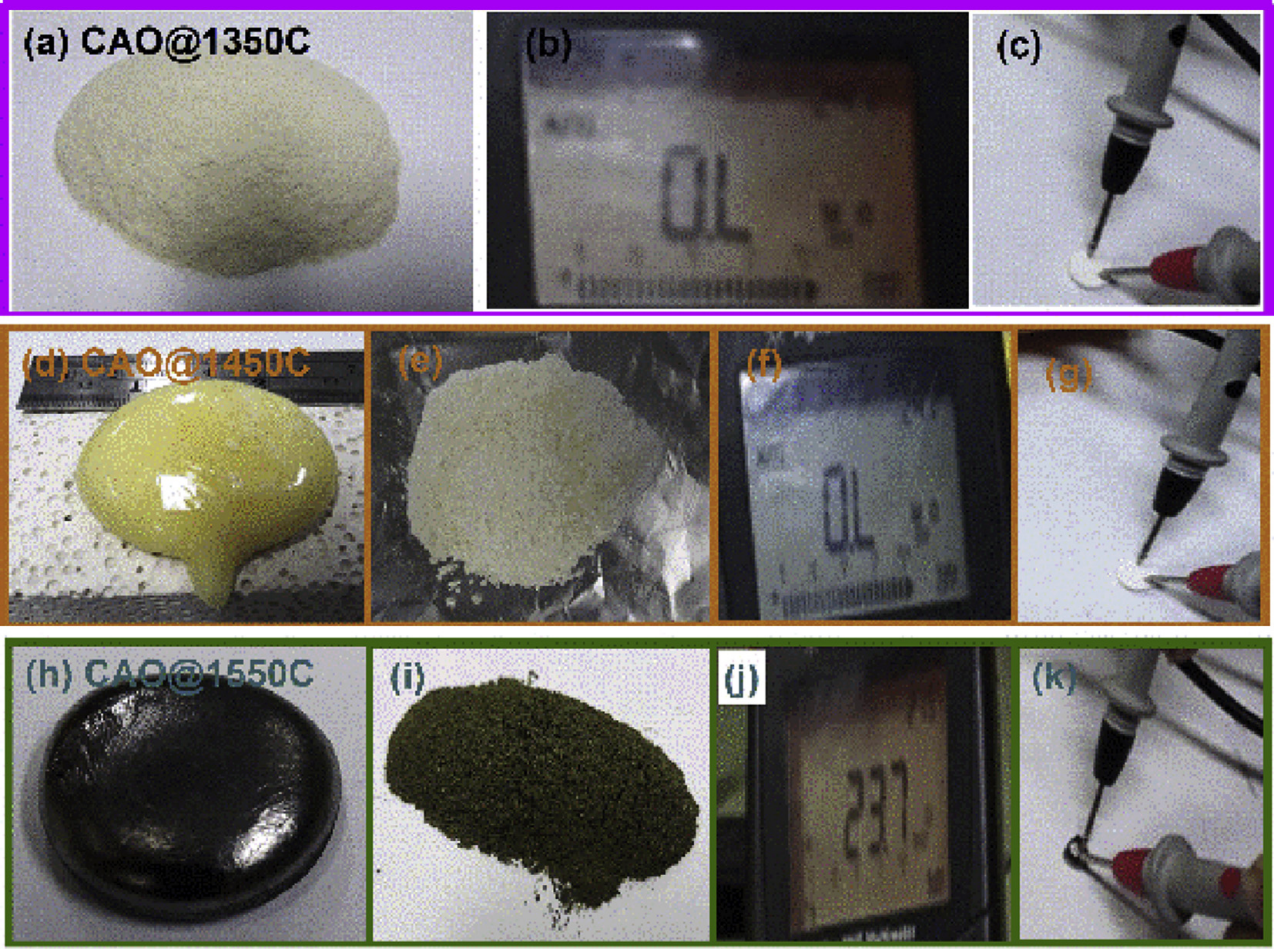
Images of synthesized CAO@1350C, CAO@1450C and CAO@1550C samples fabricated at 1350 °C, 1450 °C and 1550 °C, respectively, (a) white colored of CAO@1350C powder, (b) and (c) measurement of the electric resistances of CAO@1350C sample as presenting electric insulator using a multi-meter, (d) yellow colored of CAO@1450C sample that formed a single crystal, (e) yellow colored crushed powder of CAO@1450C sample, (f) and (g) measurement of the electric resistances of CAO@1450C sample as presenting electric insulator, (h) greenish-black colored of CAO@1550C sample with a cement from a single crystal, (i) green colored crushed of CAO@1550C powder, and (j) and (k) measurement of the electric resistances of CAO@1550C sample as presenting electric conductor.

Fig. 5(a-k) presents images CAO@1350C, CAO@1450C and CAO@1550C samples fabricated at 1350 °C, 1450 °C and 1550 °C, respectively, and measurement of their electric resistances using a multi-meter. Fig. 5 (a) shows a white colored powder sample. This result reveals that the sample did not form a single crystal phase at this temperature. The resistance of the sample was very high as shown in Fig. 5 (b) and (c). Fig. 5 (d) presents a yellow colored sample that formed from a single crystal in the crushed powder sample (Fig. 5 (e)). A single crystal phase was produced that had a very high resistance as shown in Figs. 5 (f) and (g). Fig. 5 (h) shows a greenish-black colored sample with a cement from a single crystal type forming a green colored crushed powder (Fig. 5 (i)). This sample, sintered at 1550 °C, was electrically conductive as shown in Figs. 5 (j) and (k), leading to the observation that sintering at temperatures higher than 1450 °C produced a single crystal form of Ca_12_Al_14_O_33_ cement. Only the CAO@1550C sample displayed electrical conductivity after its formation at 1550 °C. The green color of this sample was not the same as the CAO@1350C and CAO@1450C samples implying a different mechanism for fabrication of the CAO@1550C sample than the CAO@1350C and CAO@1450C samples.

#### 3.2.4 Absorption coefficients

**Fig. 6:**
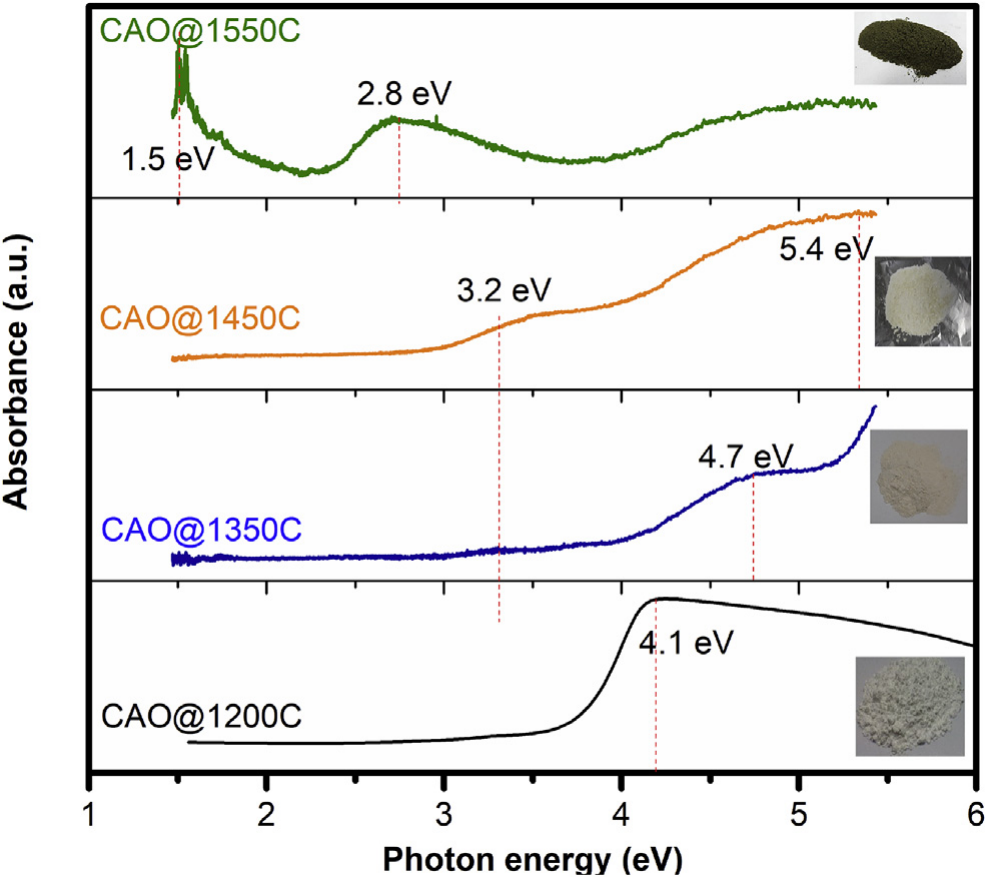
Absorption coefficient as function of photon energy of CAO@1200C, CAO@1350C CAO@1450C and CAO@1550C samples.

The differences between the CAO@1550C, CAO@1350C and CAO@1450C cement phases were investigated from their optical absorption coefficients measured using UV Visible Spectrometry. Fig. 6 shows absorption coefficients in the UV spectrum of the prepared CAO@1200C starting powder, and the synthesized CAO@1350C, CAO@1450C and CAO@1550C samples at room temperature over the range of 1.6 eV–6 eV. The absorption spectra of the prepared CAO@1200C sample had the highest absorption peak at 4.1 eV. This was characteristic of the electrically insulating Ca_12_Al_14_O_33_ cement phase with an extra-framework O_2_^2-^ (superoxide radical) and O^2-^ (oxygen anion radical). They were inside the cavity-cages of the structure, denoted as Ca_12_Al_14_O_33_:O cement. The absorption spectra of the CAO@1350C and CAO@1450C samples showed their two highest absorption peak positions at 3.2 eV and 4.7 eV for the CAO@1350C sample, and 3.2 eV and 4.7eV for the CAO@1450C sample. The absorption edge value of 3.2 eV for the CAO@1450C sample was attributed to the excitation between the energy level of encaged free oxygen O^2-^ ions and the cage conduction band (CCB) as previously reported [19]. These implied the presence of extra-framework O^2-^ ions in a nano-cavity cage within the structure of the insulating Ca_12_Al_14_O_33_:O cement, similar to the CAO@1200C sample. Additionally, the absorption spectra of the CAO@1550C sample displayed its two highest absorption peak positions at 2.8 eV and 1.5 eV. The first peak at 2.8 eV presented electrons transitioning from the occupied cage level (an F^+^-like center level due to a relaxation time) to the framework conduction band (FCB), as previously reported [28,29]. The energy at 2.8 eV was due to the inter-cage transition energy for the free electrons in cavity-cage structure. The second peak at 1.5 eV displayed an energy level from the F^+^-like center level to the cage-conduction band (CCB). A second peak energy level was reported in range of 0.4–1.5 eV [28, 29, 30, 31]. This energy level was too large for the empty cage and an electron with less energy to occupy the cage. The two peak positions at 2.8 and 1.5 eV were characteristic of conducting Ca_12_Al_14_O_33_:e^−^ cement. These results confirmed that the conducting Ca_12_Al_14_O_33_:e^−^ cement was completely converted from insulating Ca_12_Al_14_O_33_:O cement when heated inside the carbon crucible to a temperature of 1550 °C. Obviously, this method can replace free oxygen in the cavity-cage with a free electron to form conducting Ca_12_Al_14_O_33_:e^−^ cement. The absorption peaks at 1.5–2.8 eV of the CAO@1550C sample were correlated to the greenish-black color of the sample shown Fig. 5. The powder color changed from white to green comparing the CAO@1200C to CAO@1550C samples. The sample color was caused by the free electron concentration in the structure. The white and green color samples presented free electron concentrations of 0 cm^−3^ and around 1×10^20^ cm^−3^, respectively, which is good agreement with earlier reports [32]. The CAO@1200C sample, i.e., starting powder, as well as the CAO@1350C and CAO@1450C samples, displayed a light color without free electrons in the structure. The CAO@1550C powder had a green color, with approximately 2×10^20^ cm^−3^ of free electrons. Additionally, the CAO@1200C, CAO@1350C and CAO@1450C samples were electric insulators while the CAO@1550C sample was electrically conductive.

The CAO@1200C starting powder, as well as the CAO@1350C and CAO@1450C samples, were white colored, indicating that they were transparent in the visible light region (1.6–3.2 eV), as well as acting as electrical insulators. These results illustrated that the CAO@1200C, CAO@1350C and CAO@1450C samples were electric insulators as was the insulating Ca_12_Al_14_O_33_:O cement owing to free oxygen radicals in their cavity-cages. The greenish-black color of the CAO@1550C sample indicated light absorption in the region of 1.5–2.8 eV. This confirmed that sintering at 1550 °C by rapid induction heating could convert electrically insulating Ca_12_Al_14_O_33_:O cement to conducting Ca_12_Al_14_O_33_:e^−^ cement. This process can replace free oxygen radicals with free electrons in an electron-doped process inside a nano-cage Ca_12_Al_14_O_33_ cement structure.

#### 3.2.5 SEM and EDS analysis

**Fig. 7:**
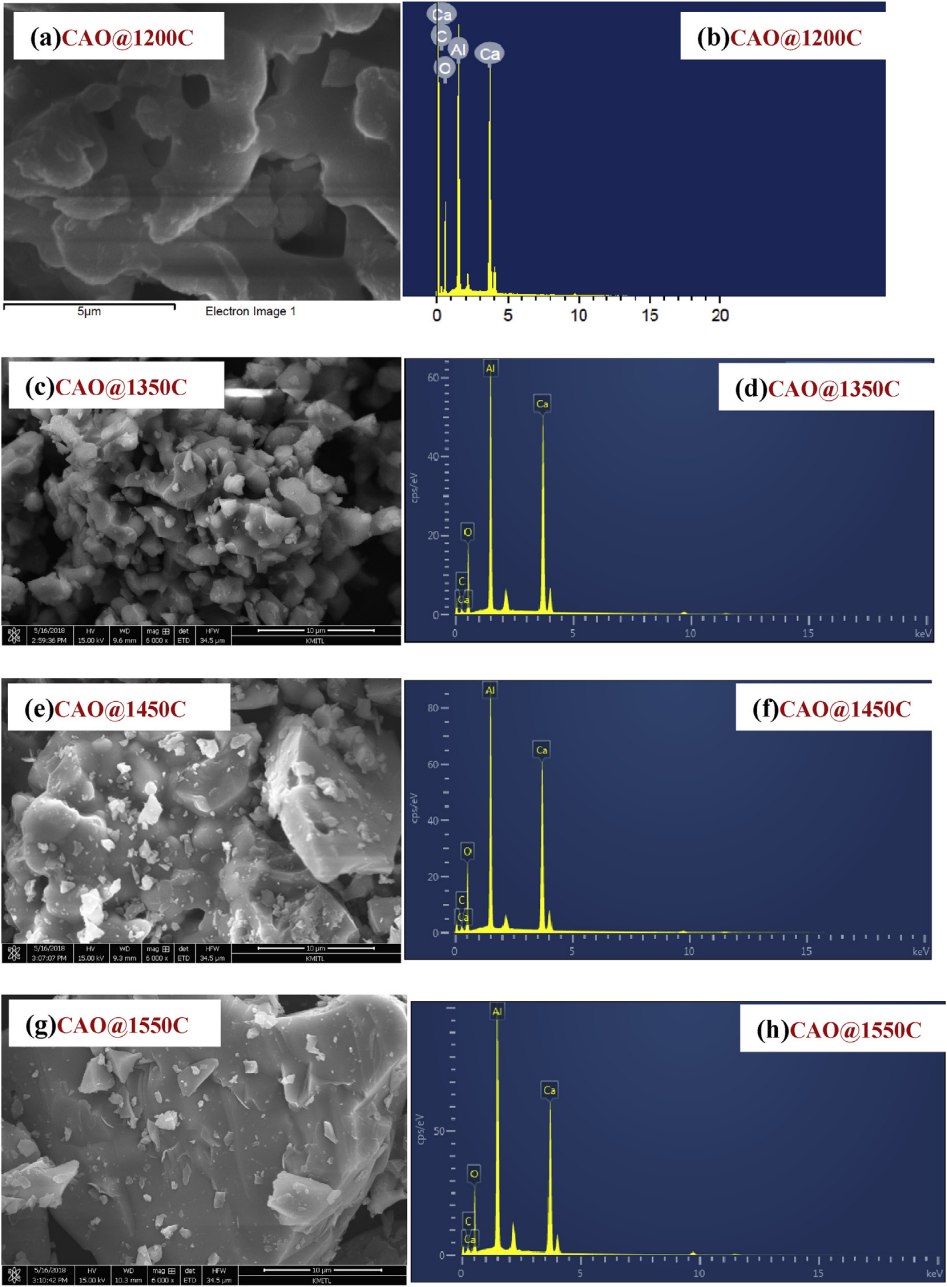
SEM (a), (c), (e) and (g), and EDX (b), (d), (f) and (h) of the synthesized CAO@1200C, CAO@1350C, CAO@1450C and CAO@1550C samples, respectively.

The imagery in Fig. 7 (a), (c), (e) and (g) show SEM analysis and Figs. 7 (b), (d), (f) and (h) show EDS mapping of the synthesized CAO@1200C, CAO@1350C, CAO@1450C and CAO@1550C samples, respectively. All of the samples presented micrometer grain sizes. The EDS results show the presence of Ca, Al, O and C atoms, indicating that the samples formed a CAO phase structure. There was a homogeneous distribution of carbon atoms on the powder surfaces. Additionally, the percentage of C atoms was similar in all samples.

**Fig. 8:**
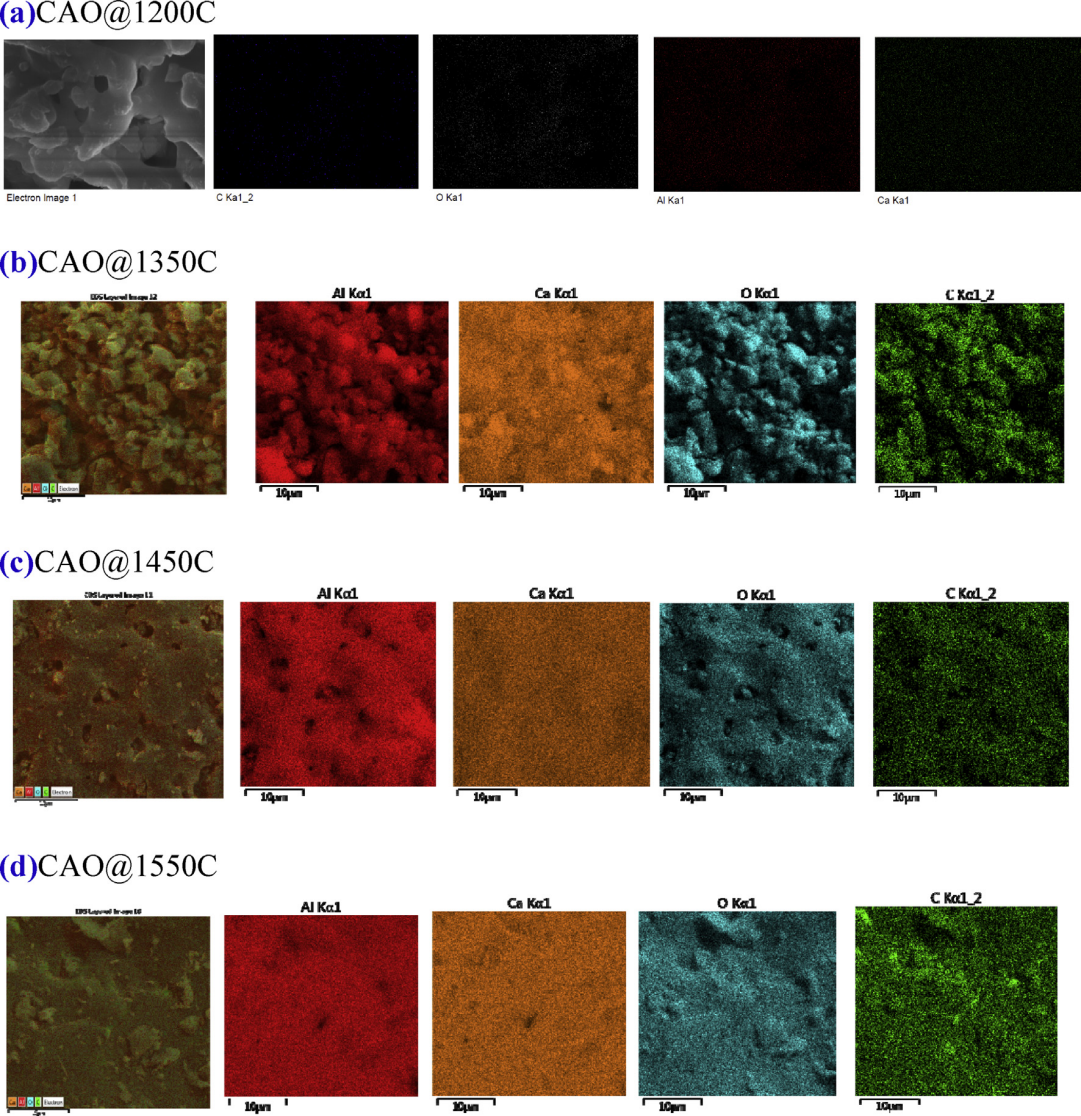
Elemental distribution (SEM-EDS) of the synthesized **(a)** CAO@1200C, **(b)** CAO@1350C, **(c)** CAO@1450C and **(d)** CAO@1550C samples with a homogeneous distribution of the Ca, Al, O and C atoms on the powder surfaces.

Thus, the results imply that the carbon remaining component of the synthesized process did not alter the percentage of carbon in the obtained CAO@1200C, CAO@1350C, CAO@1450C and CAO@1550C samples. EDS mapping of the CAO@1200C, CAO@1350C, CAO@1450C and CAO@1550C samples showed a homogeneous distribution of the Ca, Al, O and C atoms on the powder surfaces in Fig. 8 (a), (b), (c) and (d), respectively.

### 3.3 Properties of conducting Ca_12_Al_14_O_33_

#### 3.3.1 Raman spectroscopy analysis

**Fig. 9:**
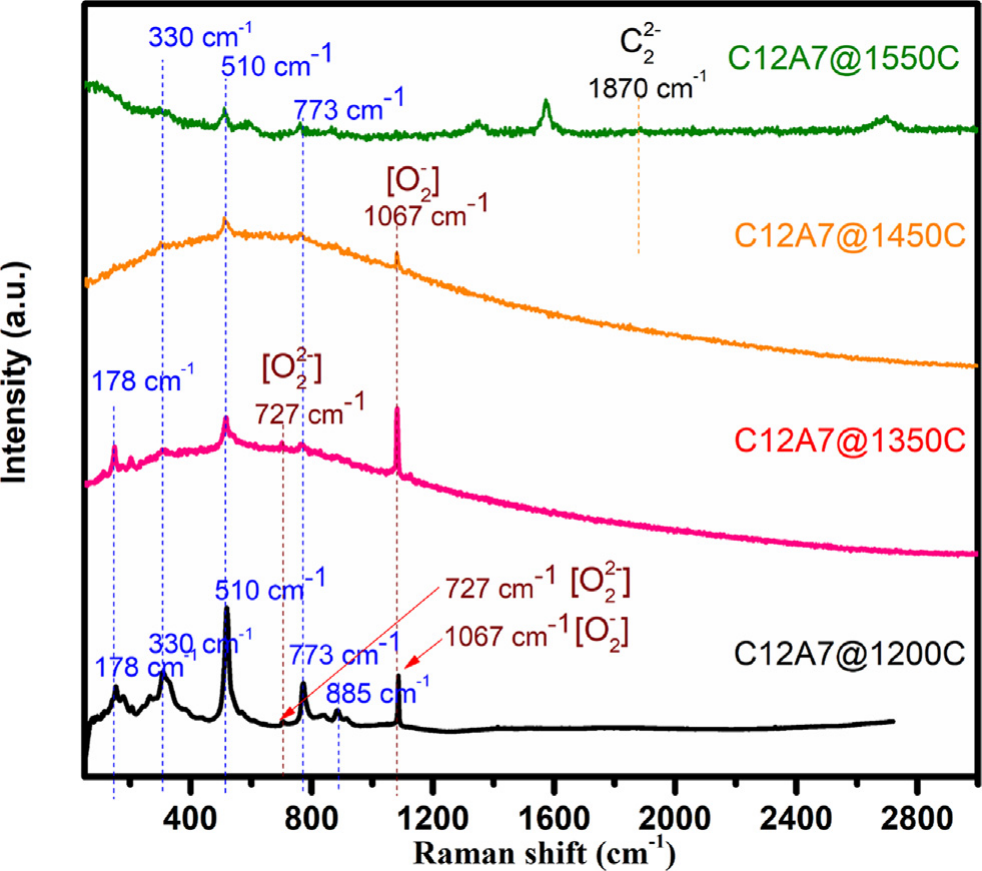
Raman spectra of the CAO@1200C sample and synthesized CAO@1350C, CAO@1450C and CAO@1550C samples.

Raman spectroscopy [34, 35, 36, 37] was used to characterize the molecular structure and bonding of the fabricated materials. Fig. 9 shows Raman spectra of the prepared CAO@1200C sample as well as the synthesized CAO@1350C, CAO@1450C and CAO@1550C samples. The absorption bands for the Ca_12_Al_14_O_33_ cement structure have normal lattice properties due to vibrations of Al^3+^, Ca^2+^ and oxygen ions in the region from 50 to 3000 cm^−1^ [37]. In previous work [37], Raman peaks between 200 and 1000 cm^−1^ were ascribed to the lattice framework of the Ca_12_Al_14_O_33_ cement structure resulting from Al^3+^ ions in a tetrahedral structure. The Raman spectra peaks at 330, 510, and 773 cm^−1^ of the CAO@1350C, CAO@1450C and CAO@1550C samples corresponded to peaks of the CAO@1200C sample. The Raman peak at 330 cm^−1^ was caused by the oxygen (O^2-^) framework due to vibrations of Ca[AlO_4_] and Ca–O bonding. The two bands at 510 cm^−1^ and 773 cm^−1^ indicated bending vibrations the Al–O–Al linkages and Al–O stretching vibrations, respectively, for a lattice structure with the oxygen and aluminum atoms in a symmetric framework with an Al–O [AlO_4_]^5-^ sub-structure [38]. The band at 178 cm^−1^ appeared on the CAO@1200C and CAO@1350C sample and was identified as characteristic of the lattice framework caused by Al^3+^ ion coordination. This peak may be characteristic of insulating Ca_12_Al_14_O_33_ cement prepared at temperatures lower than 1350 °C. The band at 885 cm^−1^ appeared only in the CAO@1200C sample resulting from bending and stretching vibrations of the Al and O framework. This peak was characteristic of insulating Ca_12_Al_14_O_33_ cement prepared at 1200 °C. The CAO@1200C sample exhibited two band peaks at around 1338 cm^−1^ and 1586 cm^−1^ that appeared only in the CAO@1500C sample resulting from a small amount of graphite from the carbon crucible that was not observed in the XRD results of Fig. 4.

Additionally, a peak at 727 cm^−1^ was observed in the CAO@1200C and CAO@1350C samples. In previous work [37], a band located at 727 cm^−1^ indicated O_2_^2−^stretching of the extra-framework O_2_^2−^ion in electrically insulating Ca_12_Al_14_O_33_ cement. The peak at 1067 cm^−1^ represents the stretching mode of the extra-framework O_2_^−^ ions of the CAO structure [37]. A peak at 1067 cm^−1^ was presented by the CAO@1200C, CAO@1350C and CAO@1450C samples, indicating the presence of extra-framework O_2_^−^ ions. The formation process of the O_2_^2−^ ions can be described by Eqs. (1) and (2) [39]:

(1) O_2_ (atmospheric) → O_2_ (cage)

(2) 2O^2−^ (cage) + O_2_ (cage) → 2O_2_^2−^ (cage)

Additionally, the formation of superoxide (O_2_^−^) ions occurred via the following chemical reaction in Eq. (3) [33]:

(3) O^2−^(cage) + O_2_ (cage) → O^−^ (cage) + O_2_^−^ (cage)

Formation of O_2_^2−^ions can be described by Eq. (4) [39]:

(4) 2O^−^ (cage) → O_2_^2−^ (cage)

These reactions indicated that the extra-framework O_2_^2−^ ions in the nano-cage cavity of the structure were present in the Ca_12_Al_14_O_33_ cement prepared in the temperature range of 1200–1350 °C. Extra-framework O_2_^−^ and O^−^ ions were present in the samples prepared at temperatures of 1200–1450 °C. Oxygen radical O_2_^−^and O^−^ions were successfully replaced by extra-framework O_2_^2−^ ions in a caged structure at 1350 °C.

Moreover, the Raman spectra bands at 725 and 1065 cm^−1^ were not observed in the CAO@1550C sample, indicating a reduction of insulating Ca_12_Al_14_O_33_ cement into a conductive form. Extra-framework O_2_^2−^, O_2_^−^ and O^−^ ions were not observed in the nano-cage cavity of the CAO@1550C sample. This implied that the CAO@1550C sample was completely converted from insulating Ca_12_Al_14_O_33_ cement to its conducting form. This mechanism successfully replaced free oxygen ions in a cavity-cage with free electrons via the reaction in Eq. (5):

(5) C_2_^2−^ (gas) + O_2_^−^ (cage) → e^−^ (cage) + CO (gas)+ CO_2_ (gas)

This mechanism required free radical carbon ions (C_2_^2−^) at a reaction temperature of 1550 °C. In this process, the C_2_^2−^ ions were generated from the carbon crucible that reacted with O_2_^−^ions when it was heated to 1550 °C. Then, the reaction removed free oxygen O_2_^−^ ions and injected free electrons (e^−1^) into the nano-cage structure. No peaks at 1870 cm^−1^ were observed for the CAO@1550C sample. Kim et al. [33] reported Raman band spectra at 1870 cm^−1^, ascribing them to C_2_^2−^ ions. C_2_^2−^ions were dissolved into the CAO@1550C sample from the C_2_^2−^ atmosphere of the carbon crucible and were released out of the nano-cages during cooling. In summary, the extra-framework O_2_^2−^ and O_2_^−^ ions present in the CAO@1200C, CAO@1350C and CAO@1450C samples were responsible for producing an insulating Ca_12_Al_14_O_33_ cement. The CAO@1550C sample, i.e., Ca_12_Al_14_O_33_:e^−^ cement, had free electrons in a nano-cage structure that allowed for electrical conduction.

#### 3.3.2 Optical properties

**Fig. 10:**
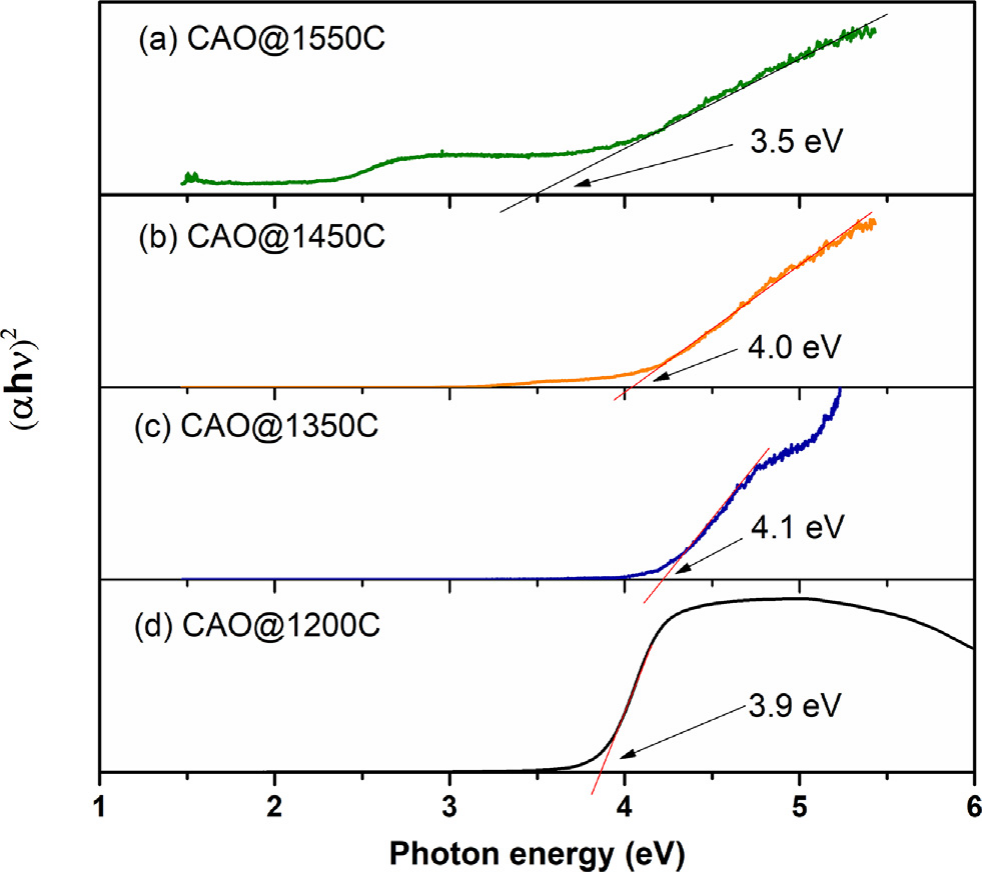
Absorption coefficient of the CAO@1200C sample, and sintered CAO@1350C, CAO@1450C and CAO@1550C samples at room temperature in range of 1.6 eV–6 eV measured by UV-Vis spectroscopy.

The absorption coefficients shown in Fig. 6 present the optical properties of the CAO@1200C, CAO@1350C, CAO@1450C and CAO@1550C samples. They were used to calculate to optical energy gap (*E*_*g*_) following the relationship in Eq. (6) [20]:

(6) $$\alpha \left(h\upsilon \right)\propto \frac{{\left(h\upsilon -E_{g}\right)}^{1/2}}{h\upsilon }$$

where *hυ* denotes the photon energy, *E*_*g*_ represents the direct optical gap, and 1/2 is a value for the allowed direct transition type. Thus, the allowed direct optical gap can be calculated from Eq. (7):

(7) $${\left(\alpha h\upsilon \right)}^{2}=A\left(h\upsilon -E_{g}\right)$$

where *A* is a constant. The optical energy gap (*E*_*g*_) was fitted to a straight line to intercept the photon energy (*hυ*) axis. Fig. 10 shows the optical gap of the prepared CAO@1200C sample, as well as the sintered CAO@1350C, CAO@1450C and CAO@1550C samples at room temperature. Their energy gap values were 3.9 eV, 4.1 eV, 4.0 eV and 3.5 eV, respectively. The results showed that the CAO@1200C, CAO@1350C, and CAO@1450C energy gap values were close to 4.0 eV, indicating the insulating nature of this Ca_12_Al_14_O_33_ cement. This value (4.0 eV) represents the energy gap for the allowed direct optical gap of the electronic transition from an occupied electronic state of the upper part of the framework conduction band (FCB) to the cage conduction band (CCB). Moreover, the energy gap of the CAO@1550C sample was 3.5eV as indicating a conductive Ca_12_Al_14_O_33_ cement phase structure. This value (3.5 eV) represents the energy of the electronic transition from the occupied framework valence band (FCB) to the cage conduction band (CCB).

#### 3.3.3 First-principles calculations

First-principles calculations for evaluating the optical properties of Ca_12_Al_14_O_33_ were performed as previously outlined [40,41]. The electronic properties of insulating Ca_12_Al_14_O_33_:2O^2-^ cement and conducting Ca_12_Al_14_O_33_:4e^−^ cement structures were determined in this manner. In the present study, the unit cell of the Ca_12_Al_14_O_33_:2O^2-^ structure contained 12 lattice framework cages [Ca_24_Al_28_O_64_]^4+^ and two O^2-^ anions (extra-framework) trapped at the center of a cage. The determination of the unit cell of Ca_12_Al_14_O_33_:e^−^ used Ca_12_Al_14_O_33_:2O^2-^ as an initial configuration and two O^2-^ anions were removed followed by the addition of four extra electrons inside the unit cell. The results showed that the lattice constants of the optimized structures were 11.8576 and 12.0256 Å for insulating Ca_12_Al_14_O_33_:2O^2-^ cement and conducting CAO: e^−^ cement, respectively.

**Fig. 11:**
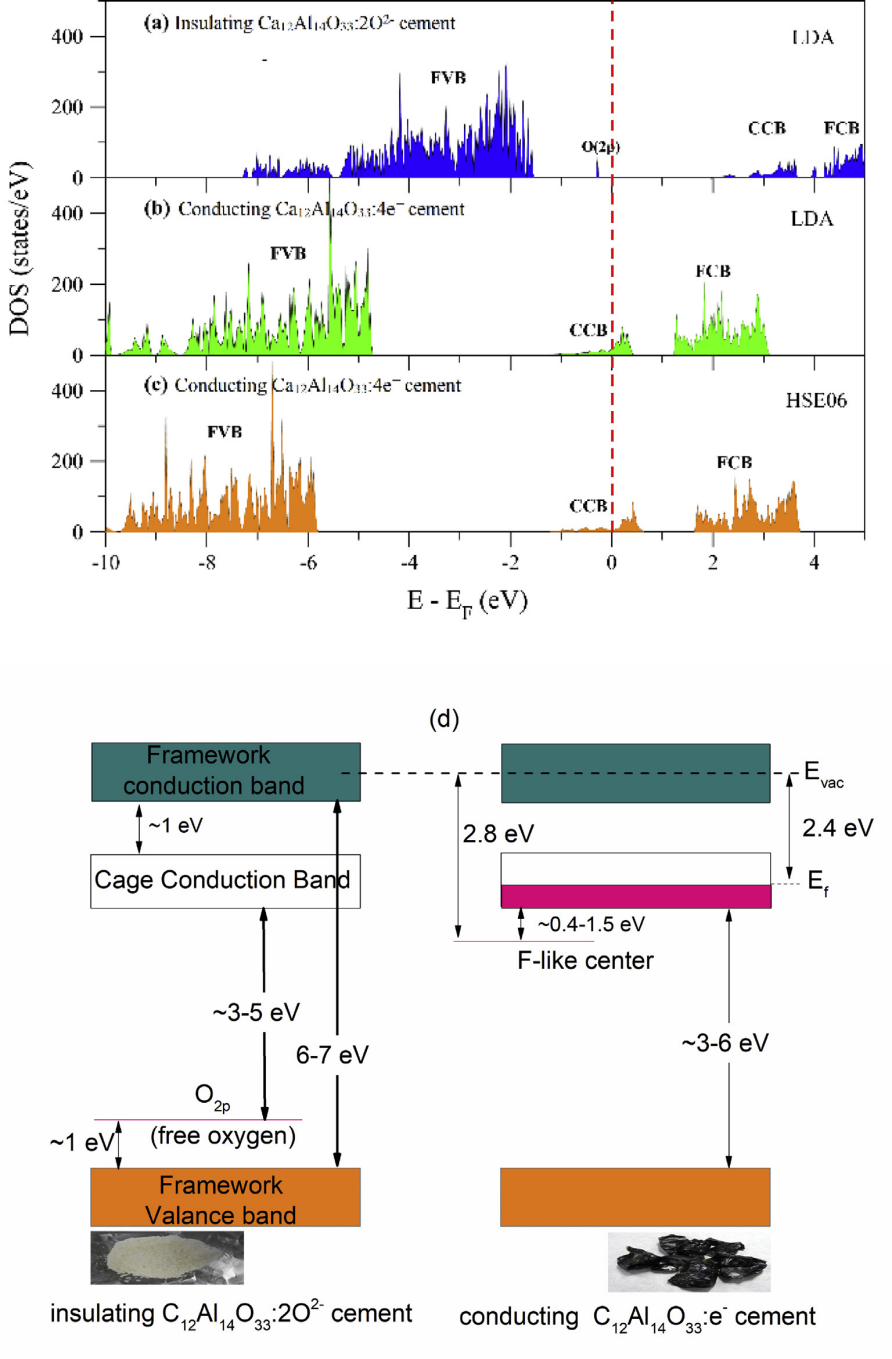
The electronic density of states of (a) Ca_12_Al_14_O_33_:2O^2-^, (b) Ca_12_Al_14_O_33_:4e^−^ calculated using the LDA functional and (c) the electronic density of states of Ca_12_Al_14_O_33_:e^−^ computed using the HSE06 functional for the framework valence band (FVB), framework conduction band (FCB) and cage conduction band (CCB), respectively, (d) energy diagram of insulating Ca_12_Al_14_O_33_:2O^2-^ mayenite and conducting Ca_12_Al_14_O_33_:4e^−^ electride, respectively.

For Ca_12_Al_14_O_33_:2O^2^, Fig. 11 (a) clearly shows a peak located at 0.3 eV below the Fermi level corresponding to the 2p states of extra-framework 2O^2-^ ions. Additionally, the energy gap in this case is the difference between the energies of the highest states of the framework valence band (FVB) and the lowest states of the CCB. In this case, we found that the energy gap is approximately 4.1 eV. This result was close to the energy gap of the experimental results for CAO@1200C (3.9 eV), CAO@1350C (4.1 eV) and CAO@1450C (4.0 eV) as shown Fig. 10. The implication of this is that the CAO@1200C, CAO@1350C and CAO@1450C samples have a Ca_12_Al_14_O_33_:2O^2-^ structure. The 4.1 eV energy gap for the Ca_12_Al_14_O_33_:2O^2-^ represents the minimum energy required for the transition from the FVB to CCB. It was found that at least 2.3 eV is required for the electronic transition from the extra-framework O^2-^ ions to the CCB. The above results confirm that the CAO@1200C, CAO@1350C and CAO@1450C samples are electrically insulating cement materials.

The electronic density of states of Ca_12_Al_14_O_33_:e^−^ was determined using the local density approximation (LDA) scheme shown in Fig. 11 (b). It is well established that electrical conductivity is directly related to the electron states at the Fermi level. A large number of electron states at the Fermi level energy, E_F_ (the highest occupied state), which is at 0 eV, results in high electrical conductivity.

According to the density of states of Ca_12_Al_14_O_33_:e^−^, it can easily be seen that there are CCB states at the Fermi level. This result implies that electron conduction comes from the CCB, in this case. Hence, Ca_12_Al_14_O_33_:e^−^ can be electrically conductive. However, the energy gap obtained from LDA functional analysis was always smaller than the experimentally derived energy gap. To improve the accuracy of our calculations, a hybrid functional was used. For Ca_12_Al_14_O_33_:4e^−^, we determined the electronic density of states using the Heyd-Scuseria-Ernzerh of 06 (HSE06) hybrid functional shown in Fig. 11 (c). The electronic density of states calculated from the HSE06 hybrid functional looks similar to that determined from the LDA functional. Moreover, we found that the FVB states shifted to slightly lower energy levels relative to the FVB of LDA. Hence, the energy gap increased in this case. The energy difference between the highest FVB state and the lowest CCB state was approximately 4.7 eV. Additionally, the energy required for the electronic transition from FVB to FCB was rather high, 7.4 eV. Thus, it is impossible for electrons to be excited from the FVB to the FCB.

The energy difference between the highest FVB state and the lowest CCB state was approximately 3.7 eV–4.7 eV. This result was close to the experimentally derived energy gap for CAO@1550C (3.5 eV). The energy gap between FCB and CCB was about 0.42 eV, representing the metallic bands of Ca_12_Al_14_O_33_:e^−^ that are characteristic of the cage-like structures with no extra-framework oxygen species.. According to Fig. 11 (b) and (c), only electron states of the CCB exist at the Fermi level. Consequently, electronic conduction was observed in this material because of the free electrons in the cage. The above results are consistent with previous work [42,43]. From Figs. 6, 10, and 11, the energy diagram for insulating Ca_12_Al_14_O_33_:2O^2-^ and conducting Ca_12_Al_14_O_33_:e^−^ cements obtained from both experiments and calculations [28,30,43] is summarized in Fig. 11(d).

Moreover, the experimental and calculation results showed that the insulating Ca_12_Al_14_O_33_:2O^2-^ cement had a direct optical gap of approximately 4.0 eV for the electronic transition from the FVB to the CCB. For conducting Ca_12_Al_14_O_33_:e^−^, the experimental and calculational results showed the direct transition gap from FVB to CCB was around 3.5–3.7 eV. This information indicated that it is impossible for electron excitations to occur between the FVB to FCB due to a rather large energy gap (3.5 eV). The conducting Ca_12_Al_14_O_33_:e^−^ cement displayed metallic behavior when it absorbs external energy at around 2.8 eV, corresponding to the energy level from CCB to FCB for free electron movement between cages. This energy (2.8 eV) corresponds to the absorption peak position at 2.8 eV that results in the greenish-black color of the sintered CAO@1550C sample shown in Fig. 5. Additionally, electrons become free electrons when their energy becomes greater than the work function energy (E_vac_), which is 2.4 eV [31,44]. The broad absorption band at 2.8 eV for the CAO@1550C sample is attributed to the transition to intra-cage s-to-p forms of electrons trapped in the cages. The absorption band at 1.5 eV was due to the transition of inter-cage s-to-s charge transfer from an electron trapped in a cage to a vacant neighboring cage [38]. The transition of free electron movement of the CAO@1550C sample (Ca_12_Al_14_O_33_:e^−^ cement) was supported by oxygen gas adsorbed at the surfaces of the materials to produce free radicals [45] according to the reaction Eq. (8):

(8) O_2_(gas) + e^−^(cage) → O_2_^-^

### 3.4 Schematic diagram for fabrication of conducting Ca_12_Al_14_O_33_

**Fig. 12:**
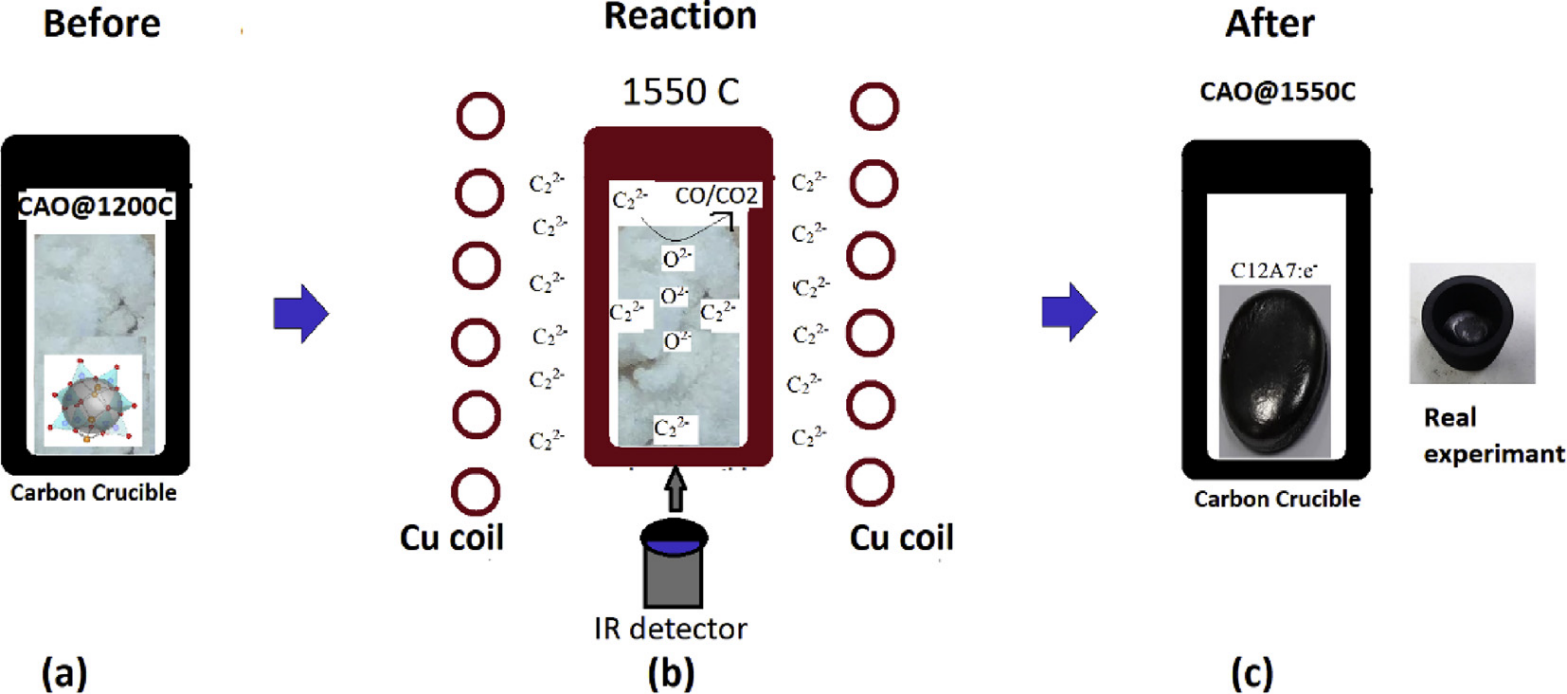
Schematic diagram of the synthesized conducting Ca_12_Al_14_O_33_:e^−^ cement fabricated from insulating Ca_12_Al_14_O_33_:O^2-^ cement using a carbon crucible heated by a Cu induction coil via high frequency electromagnetic induction heating, (a) Ca_12_Al_14_O_33_:O^2-^ (CAO@1200C) cement powder in a carbon crucible, (b) the process of replacing of free oxygen ions by free electrons at powder surfaces via a reaction of active carbon (C_2_^2-^) with free oxygen ions in a cavity-cage to produce CO/CO_2_ gas at 1550 °C and (c) the formation of conducting Ca_12_Al_14_O_33_:e^−^ cement.

A conducting Ca_12_Al_14_O_33_:e^−^ cement was successfully fabricated via a sintering process starting with insulating Ca_12_Al_14_O_33_:O^2-^ cement inside a carbon crucible using high frequency induction heating to 1550 °C. A schematic presenting the mechanism for converting insulating Ca_12_Al_14_O_33_:O^2-^ cement to conducting Ca_12_Al_14_O_33_:e^−^ cement via this process is shown in Fig. 12. Fig. 12(a) shows the starting Ca_12_Al_14_O_33_:O^2-^ cement powder loaded inside a carbon crucible. The Ca_12_Al_14_O_33_:O^2-^ structure is a linkage of calcium, aluminum and oxygen atoms consisting of empty nano-sized cages containing of free oxygen ions (O^−2^), loosely bound to a lattice framework, inside cages as an extra-framework. Fig. 12(b) presents the process for producing conducting Ca_12_Al_14_O_33_:e^−^ cement by rapid heating carbon via electromagnetic induction heating to 1550 °C in a carbon crucible. The thermal energy (*Q*) in the carbon crucible was obtained from eddy currents and induced electromagnetic power [46] via the relationship Eq. (9):

(9) *Q = σ(T)[(-1)ωB]*^*2*^

where *σ(T)* is electrical conductivity as a function of temperature(S/m), *ω* is angular frequency (rad/sec), and *B* is the magnetic vector potential. At 1550 °C, an active carbon radical (C_2_^2−^) species was generated from the heated carbon for producing a C_2_^2−^ atmosphere inside the crucible [31,33,34]. Then, the C_2_^2−^ions reacted with free oxygen ions in the cavity-cages of the Ca_12_Al_14_O_33_:O^2-^ cement powder to produce CO or CO_2_ gas, leaving an electron in the cavity-cage [34].

This process replaced free oxygen ions with free electrons by reacting active carbon C_2_^2-^ ions with free oxygen ions. First, carbon C_2_^2−^ions were produced inside the crucible. Then, the C_2_^2−^ gas reacted with free oxygen species (O^2-^) in cavity-cage at the surface layer of the insulating Ca_12_Al_14_O_33_:O^2-^ cement powder to produce CO or CO_2_ gas and ejected free electrons into the cavity-cages via the reaction in Eq. (10),

(10) C_2_^2−^ + O^2−^ → 2e^−^ + CO/CO_2_

This resulted in formation of two free electrons in the cage obtained from O^2−^ions. Finally, the reaction ran to completion when the insulting Ca_12_Al_14_O_33_:O^2-^ cement was entirely converted to conducting Ca_12_Al_14_O_33_:e^−^. Fig. 12 (c) illustrates the formation of conducting Ca_12_Al_14_O_33_:e^−^ cement. A key to the production of the Ca_12_Al_14_O_33_:e^−^ cement at 1550 °C was the active carbon (C_2_^2−^) atmosphere inside the carbon crucible at high temperature [34].

### 3.5 Anti-bacterial activities of Ca_12_Al_14_O_33_ cements

**Fig. 13:**
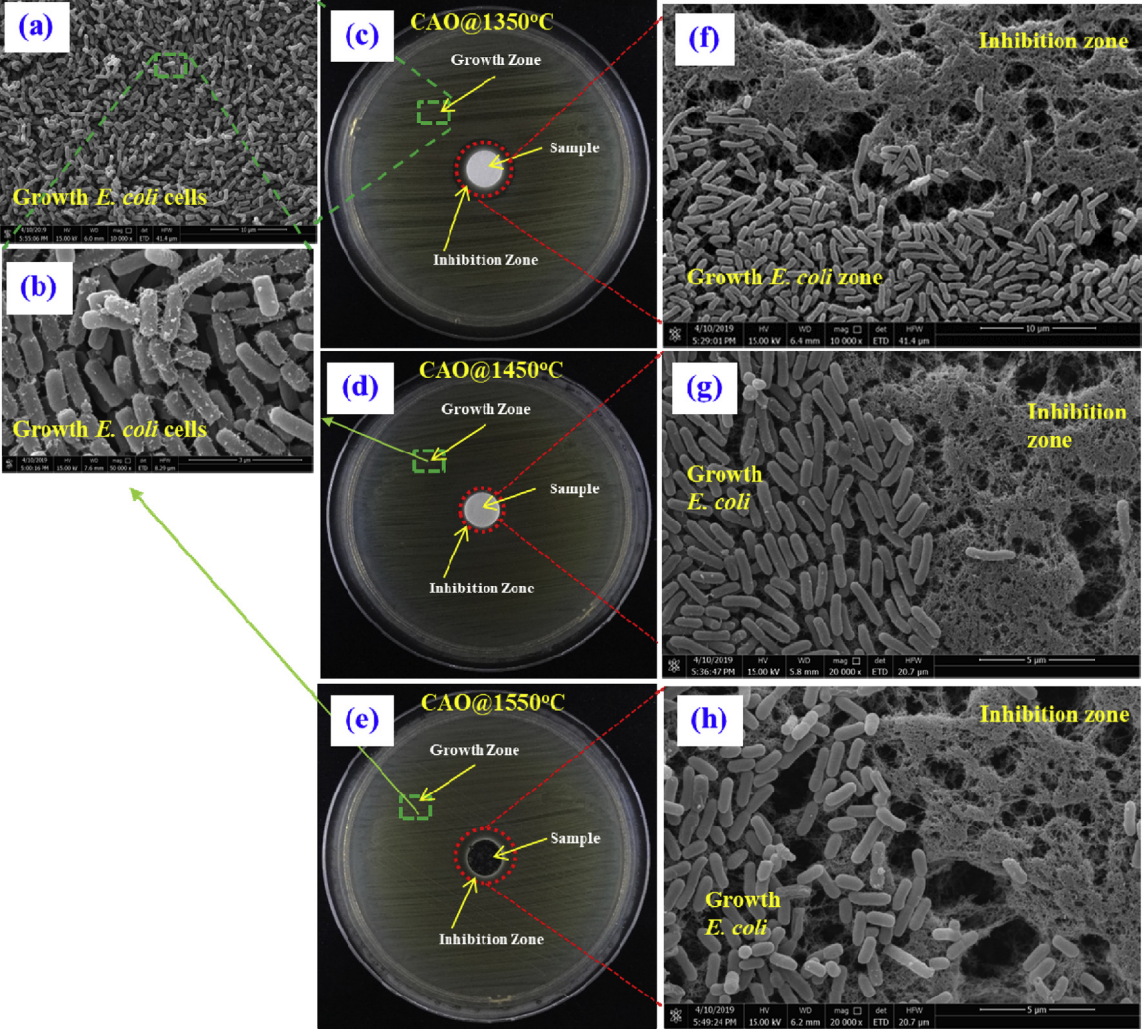
Anti-bacterial activity testing of ***E***. ***coli* (ATCC 25922)** using an agar disk-diffusion method producing zones of inhibition and SEM images showing zones of growth and inhibition of *E*. *Coli*. (a) and (b) SEM of *E*. *coli* cell grown near the CAO@1350C, CAO@1450C and CAO@1550C samples, (c), (d) and (e) inhibition of bacterial growth zones of clearing around the samples after placed on agar plates seeded with *E*. *coli* cells of the CAO@1350C, CAO@1450C and CAO@1550C, respectively, and (f), (g) and (h) SEM images of transition regions between areas with bacterial growth and inhibition of *E*. *coli* cell near the CAO@1350C, and CAO@1550C samples, respectively.

**Fig. 14:**
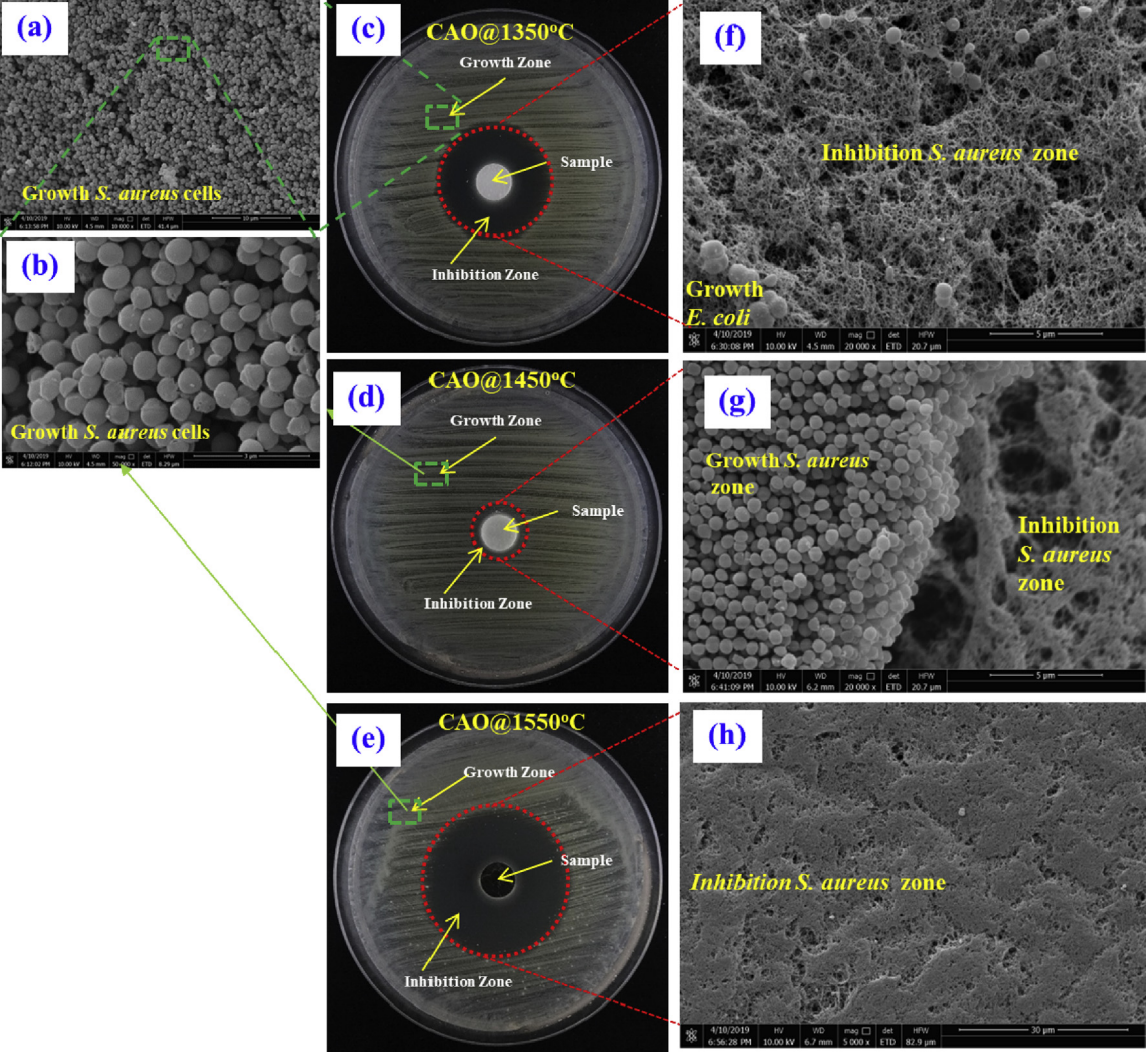
Anti-bacterial activity testing of ***S***. ***aureus* (ATCC 25923)** using an agar disk-diffusion method producing zones of inhibition and SEM images showing zones of growth and inhibition of *S*. *Aureus*, (a) and (b) SEM of *S*. *aureus* cell grown near the CAO@1350C, CAO@1450C and CAO@1550C samples, (c), (d) and (e) inhibition of bacterial growth zones of clearing around the samples after placed on agar plates seeded with *S*. *aureus* cell of the CAO@1350C, CAO@1450C and CAO@1550C, respectively, and (f), (g) and (h), SEM images of transition regions between areas with bacterial growth and inhibition of *S*. *aureus* cells near the CAO@1350C, and CAO@1550C samples, respectively.

Anti-bacterial activities of the as-fabricated CAO@1350C, CAO@1450C and CAO@1550C samples were investigated using two different bacterial species: (1) gram-negative *E*. *coli* and (2) gram-positive *S*. *Aureus* as shown in Figs. 13 and 14, respectively. The antibacterial properties of the samples were tested using an agar disk-diffusion method. Agar plates were first seeded with the test bacteria, followed by placed on the agar surfaces in a dark incubation chamber at 37 °C for 24 h. Both the insulating Ca_12_Al_14_O_33_:O^2-^ (CAO@1350C and CAO@1450C) and the conducting Ca_12_Al_14_O_33_:e^−^ (CAO@1550C) cement group materials were tested for antibacterial activity. Inhibition of bacterial growth was indicated by zones of clearing around the samples after they were placed on agar plates seeded with *E*. *coli* and *S*. *aureus* cells. SEM was used to show *E*. *coli* and *S*. *aureus* grown near the CAO@1350C, CAO@1450C and CAO@1550C samples in Figs. 13 (a) and (b), and Figs. 14 (a) and (b), respectively. The results of these tests are shown in Figs. 13 (c), (d) and (e), and Figs. 14 (c), (d) and (e), for the CAO@1350C, CAO@1450C and CAO@1550C samples, respectively. SEM images of transition regions between areas with bacterial growth and inhibition of *E*. *coli* and *S*. *aureus* cells near the CAO@1350C, and CAO@1550C samples are shown Figs. 13 (f), (g) and (h), and Figs. 14 (f), (g) and (h), respectively.

**Fig. 15:**
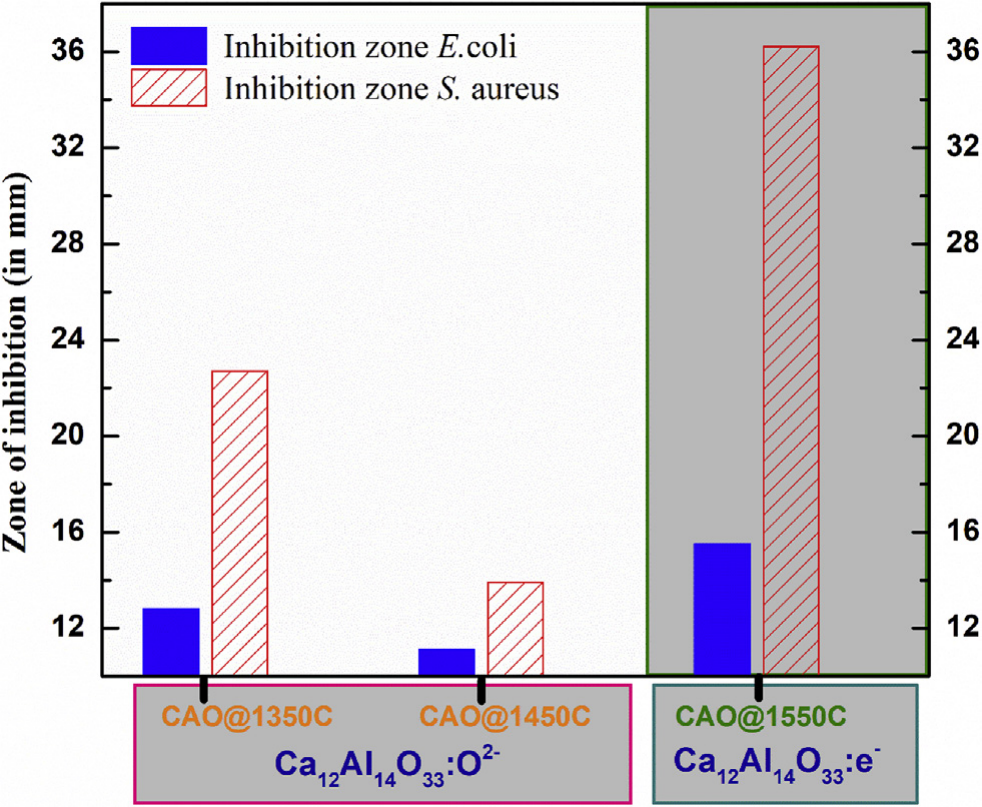
Histogram depicting inhibition zone width for insulating Ca_12_Al_14_O_33_:O^2-^ cements (CAO@1350C and CAO@1450C), and Ca_12_Al_14_O_33_:e^−^ cement (CAO@1550C) against *E*. *coli* and *S*. *aureus*.

The results from Figs. 13 and 14 demonstrate zones of inhibitions in the red-circled regions around the cement pellets. The inhibition zone widths are an index of *E*. *coli* inhibition. These zone widths were 12.8 mm, 11.1 mm and 15.5 mm for CAO@1350C, CAO@1450C and CAO@1550C samples, respectively. For *S*. *aureus*, the zones of inhibition were 22.7 mm, 13.9 mm and 36.2 mm wide for CAO@1350C, CAO@1450C and CAO@1550C samples, respectively. The histogram in Fig. 15 presents this data graphically. The results show that conducting Ca_12_Al_14_O_33_:e^−^ (CAO@1550C) exhibited much greater inhibition against both *E*. *coli* and *S*. *aureus* than the insulating Ca_12_Al_14_O_33_:O^2-^ (CAO@1350C, CAO@1450C) cement samples. The CAO@1450C cement displayed the smallest zone of inhibition resulting from its glass crystal phase (as shown in XRD resultscausing low antibacterial activity and few active sites on the sample surfaces. Moreover, it can be observed that the inhibition zones of *S*. *aureus* were larger than for *E*. *coli* due to the structural differences in the bacterial cell walls. *E*. *coli* and other gram-negative bacteria have an outer cell membrane [47,48]. This provides *E*. *coli* with more resistance to reactive oxygen species (ROS). The SEM results show rod and spherical-shaped cells in the growth zones of *E*. *coli* and *S*. *aureus* in Figs. 13 (a) and (b), and Figs. 14 (a) and (b), respectively. Neither *E*. *coli* nor *S*. *aureus* grew in the inhibition zones shown in the SEM images (Figs. 13 (f), (g), (h), and Figs. 14 (f), (g) and (h), respectively). These results confirm that both bacterial species were inhibited to varying degrees by the CAO@1350C, CAO@1450C and CAO@1550C cements.

The mechanism to inactivate these bacteria was likely due to the effect of reactive oxygen species (ROS) such as super oxides (O_2_^−^), superoxide radicals (O_2_^•-^), hydroxyl radicals (OH^•^) and hydrogen peroxide (H_2_O_2_). Several studies reported that reactive ROS interacting with bacterial cell components (proteins, lipids and DNA) cause cell death [[4], [7], [8], [9], [10], [11]]. The experimental results indicate that insulating Ca_12_Al_14_O_33_:O^2-^ cement material contained oxygen radicals (O_2_^−^and O^−^ ions), while the conducting Ca_12_Al_14_O_33_:e^−^ cement material had a high concentration of the free electrons in its structure. It follows that these two material groups had different mechanisms for bacterial inactivation.

In case of the insulating Ca_12_Al_14_O_33_:O^2-^ material, the Raman spectroscopy in Fig. 9 for the insulating Ca_12_Al_14_O_33_:O^2-^ samples (CAO@1350C and CAO1450C) displayed a band of the ROS O_2_^−^ species at 727 cm^−1^, indicating an extra-framework for oxygen radicals (O_2_^−^and O^−^ions) with concentrations up to 2 × 10^20^ cm^−1^ [24]. Clearly, the presence O_2_^−^ species at the surface of the samples can cause inactivation of *E*. *coli* and *S*. *aureus* cells, as reported earlier [24], and this mechanism requires no photocatalytic effect.

**Fig. 16:**
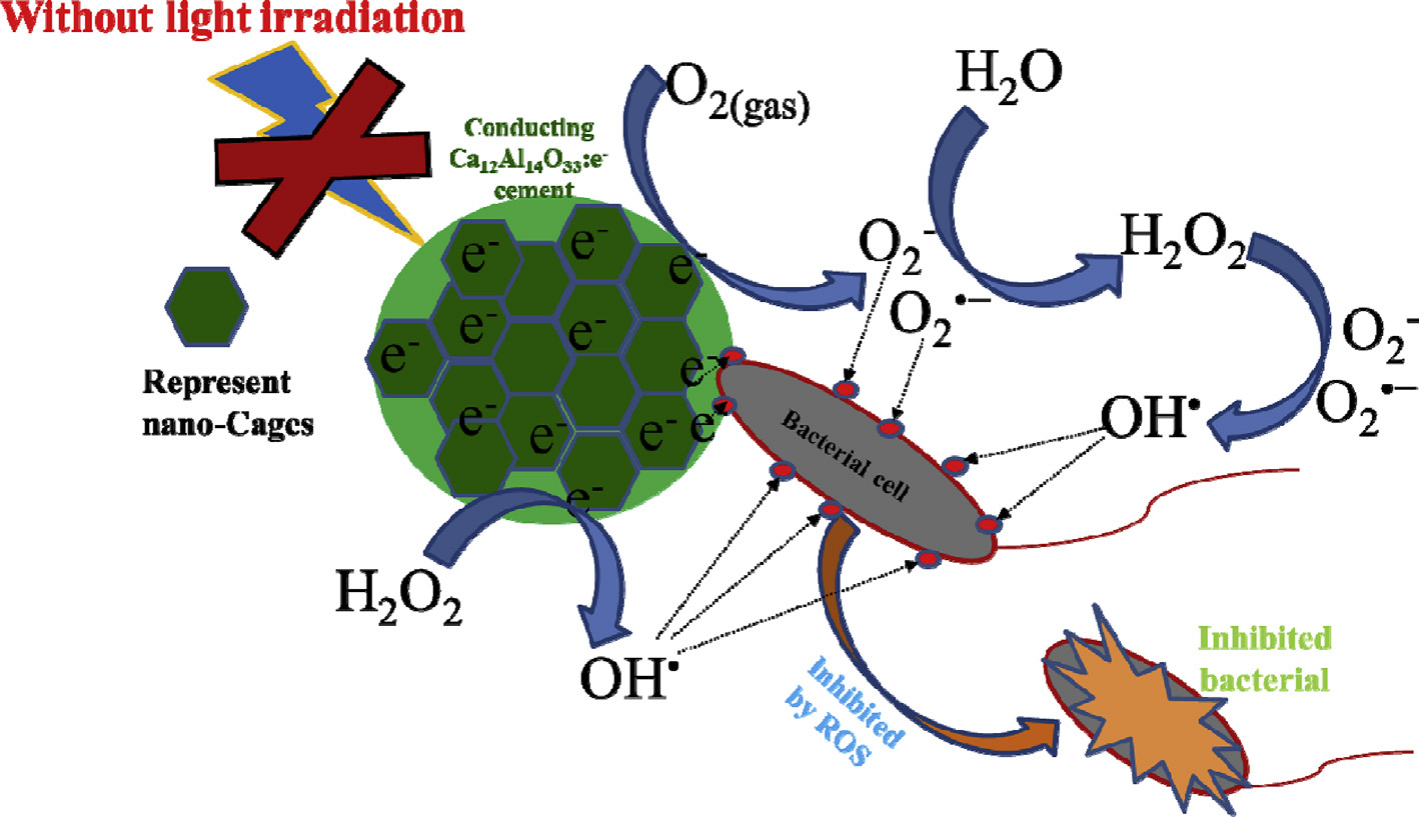
Schematic diagram of the mechanism of anti-bacterial activity of the conducting Ca_12_Al_14_O_33_:e^−^ cement.

In the case of conducting Ca_12_Al_14_O_33_:e^−^, as shown in Fig. 9, the CAO@1550C sample did not exhibit Raman bands of the ROS, O_2_^−^ and O^−^ions, species. The experimental results revealed that the CAO@1550C samples presented conducting materials with free electrons in its structure. This implies that the mechanism of bacterial inhibition of the conducting Ca_12_Al_14_O_33_:e^−^ cement was different than that of insulating Ca_12_Al_14_O_33_:O^2-^ cement. Additionally, due to the high free electron concentration (1 × 10^20^ cm^−1^) in the conducting Ca_12_Al_14_O_33_:e^−^ structure with no photocatalytic effect, free electrons at surface of the material actively reacted with atmospheric oxygen, forming superoxide anions (O_2_^−^). Then, the O_2_^−^ anions generated ROS species, i.e., H_2_O_2,_ O_2_^−^, O_2_^•−^ and OH^•^ as depicted in Fig. 16. These active oxygen species are extremely reactive, oxidizing and decomposing the organic substances of the bacteria. This implied that the conducting Ca_12_Al_14_O_33_:e^−^ cement displayed antibacterial activity with no photocatalytic effect. The reaction generating the ROS species occurred via redox reactions [8] as follows Eqs. (11), (12), (13), (14) and (15):

(11) CAO(e^−^) + O_2(gas)_ → O_2_^−^

(12) 2H_2_O + 2O_2_^−^ → 2H_2_O_2_ + O_2(gas)_

(13) CAO(e^−^) + H_2_O_2_ → OH^•^ + OH^−^

(14) H_2_O_2_ + O_2_^−^→ OH^•^ + O_2_^•−^ + OH^−^

(15) H_2_O_2_ + O_2_^•−^→ OH^•^ + O_2_ + OH^−^

Superoxide anions (O_2_^−^) were first generated from O_2_ gas in the atmosphere reacting with caged free electrons (Ca_12_Al_14_O_33_:e^−^ (cage)) at the surface of conducting Ca_12_Al_14_O_33_:e^−^ cement following Eq. (11). Then, H_2_O_2_ was generated as depicted in Eq. (12). H_2_O_2_ reacted with caged free electrons in conducting Ca_12_Al_14_O_33_:e^−^ cement, along with O_2_^−^, and O_2_^•−^ to generate OH^•^ radicals according to the reactions in Eqs. (13), (14), and (15). In this manner, conducting Ca_12_Al_14_O_33_:e^−^ cement inhibited both *E*. *coli* and *S*. *aureus* through the action of nano-caged free electrons in (Ca_12_Al_14_O_33_:e^−^ (cage). Fig. 16 schematically shows a proposed mechanism for the conducting Ca_12_Al_14_O_33_:e^−^ cement's antibacterial activity. In summary, in the first mechanism of antibacterial activity, bacteria near the surface of this material were inactivated by ROS adsorbed on the surface of the Ca_12_Al_14_O_33_ cement. The second mechanism of antibacterial activity involved bacterial inactivation by ROS species that diffused from the Ca_12_Al_14_O_33_ cement surfaces into the agar. In this way, bacteria not in contact with Ca_12_Al_14_O_33_ cement surfaces were inactivated.

Bacterial inhibition consisted of two modes, 1) the effects of ROS in the insulating cement samples, and 2) the effect of caged free electrons in the conduction cement sample. Ca_12_Al_14_O_33_:O^2-^ material displayed O_2_^-^ species at the surface of the samples causing inactivation of *E*. *coli* and *S*. *aureus* cells. Ca_12_Al_14_O_33_:e^−^ cement displayed free electrons in cavity-cages with easily movement to the surface of the material. Thus, the free electrons at surface produced ROS species, i.e., H_2_O_2,_ O_2_^-^, O_2_^•-^ and OH^•^ at the surfaces of cement materials. Testing confirmed that the Ca_12_Al_14_O_33_:e^−^ cement displayed antibacterial action against *E*. *coli* and *S*. *aureus*. Neither effect involved photocatalytic activity. These cement materials can be used in smart antibacterial walls for operation rooms and hospital wards, restaurants, nurseries, and in homes. There are also applications in HVAC and food processing.

## 4 Conclusions

Antibacterial Ca_12_Al_14_O_33_ material was successfully prepared by a rapid heating of insulating Ca_12_Al_14_O_33_ powder in a carbon crucible to a high temperature by high frequency electromagnetic induction heating. The CAO@1550C (Ca_12_Al_14_O_33_:e^−^) cement sample formed a conducting phase in the Ca_12_Al_14_O_33_ material that was confirmed by XRD results. EDX results presented the chemical composition of the Ca_12_Al_14_O_3_ cement and the UV-Vis spectroscopy showed an absorption energy at 2.8 eV, characteristic of conducting Ca_12_Al_14_O_33_:e^−^ cement. Raman spectroscopy revealed that ROS of O_2_^−^ species did not appear in the CAO@1550C sample, but was present in the CAO@1200C, CAO@1350C and the CAO@1450C samples. First principles calculations confirmed experimental results for the CAO@1550C sample. It formed a phase of conducting Ca_12_Al_14_O_33_:e^−^ cement with free electrons in a nano-cage structure of the conducting Ca_12_Al_14_O_33_ cement. The conducting Ca_12_Al_14_O_33_:e^−^ cement displayed the highest antibacterial activity against both *E*. *coli* and *S*. *aureus* with no photocatalytic activity. The antibacterial mechanism of conducting Ca_12_Al_14_O_33_ (from free electrons in a nano-caged structure) was higher for both *E*. *coli* and *S*. *aureus* than that of the insulating Ca_12_Al_14_O_33_ cement (from presence of ROS species in the structure). The inhibition of *E*. *coli* and *S*. *aureus* was due to the presence free electrons reacting with O_2_ at the surface of the materials to produce to produce ROS such as H_2_O_2,_ O_2_^−^, O_2_^•−^ and OH^•^ with no nano-sized particle interaction or photocatalytic effects. This investigation revealed that Ca_12_Al_14_O_33_ cement has antibacterial properties that can inactivate *E*. *coli* and *S*. *aureus*.

## Declarations

### Author contribution statement

Chaiwat Phrompet: Performed the experiments; Analyzed and interpreted the data.

Chaval Sriwong: Performed the experiments.

Pornjuk Srepusharawoot: Analyzed and interpreted the data.

Santi Maensiri, Prinya Chindaprasirt: Conceived and designed the experiments.

Chesta Ruttanapun: Conceived and designed the experiments; Performed the experiments; Wrote the paper.

### Funding statement

C. Ruttanapun was supported by the Thailand Research Fund (Contract Number: MRG6080236).C. Phrompet, C. Sriwong, and C. Ruttanapun were supported by the Thailand Research Fund (Contract Number: PHD60I0046). S. MAesnsiri was supported by the SUT-COE on Advanced Functional Materials, Suranaree University of Technology, Thailand. C. Ruttanapun and P. Chindaprasirt were supported by the Thailand Research Fund (TRF Distinguished Research Professor Grant No. DPG6180002).

### Competing interest statement

The authors declare no conflict of interest.

### Additional information

No additional information is available for this paper.
